# Association of Genetic Variant at Chromosome 12q23.1 With Neuropathic Pain Susceptibility

**DOI:** 10.1001/jamanetworkopen.2021.36560

**Published:** 2021-12-02

**Authors:** Abirami Veluchamy, Harry L. Hébert, Natalie R. van Zuydam, Ewan R. Pearson, Archie Campbell, Caroline Hayward, Weihua Meng, Mark I. McCarthy, David L. H. Bennett, Colin N. A. Palmer, Blair H. Smith

**Affiliations:** 1 Division of Population Health and Genomics, Ninewells Hospital and Medical School, University of Dundee, Dundee, United Kingdom; 2 Wellcome Centre for Human Genetics, University of Oxford, Oxford, United Kingdom; 3 Generation Scotland, Centre for Genomics and Experimental Medicine, Institute of Genetics and Cancer, University of Edinburgh, Edinburgh, United Kingdom; 4 Medical Research Council Human Genetics Unit, Medical Research Council Institute of Genetics and Cancer, University of Edinburgh, Edinburgh, United Kingdom; 5 Oxford Centre for Diabetes, Endocrinology, and Metabolism, University of Oxford, Oxford, United Kingdom; 6 Nuffield Department of Clinical Neurosciences, University of Oxford, Oxford, United Kingdom

## Abstract

**Importance:**

Neuropathic pain (NP) has important clinical and socioeconomic consequences for individuals and society. Increasing evidence indicates that genetic factors make a significant contribution to NP, but genome-wide association studies (GWASs) are scant in this field and could help to elucidate susceptibility to NP.

**Objective:**

To identify genetic variants associated with NP susceptibility.

**Design, Setting, and Participants:**

This genetic association study included a meta-analysis of GWASs of NP using 3 independent cohorts: ie, Genetics of Diabetes Audit and Research in Tayside Scotland (GoDARTS); Generation Scotland: Scottish Family Health Study (GS:SFHS); and the United Kingdom Biobank (UKBB). Data analysis was conducted from April 2018 to December 2019.

**Exposures:**

Individuals with NP (ie, case participants; those with pain of ≥3 months’ duration and a *Douleur Neuropathique en 4 Questions* score ≥3) and individuals with no pain (ie, control participants) with or without diabetes from GoDARTS and GS:SFHS were identified using validated self-completed questionnaires. In the UKBB, self-reported prescribed medication and hospital records were used as a proxy to identify case participants (patients recorded as receiving specific anti-NP medicines) and control participants.

**Main Outcomes and Measures:**

GWAS was performed using linear mixed modeling. GWAS summary statistics were combined using fixed-effect meta-analysis. A total of 51 variants previously shown to be associated with NP were tested for replication.

**Results:**

This study included a total of 4512 case participants (2662 [58.9%] women; mean [SD] age, 61.7 [10.8] years) and 428 489 control participants (227 817 [53.2%] women; mean [SD] age, 62.3 [11.5] years) in the meta-analysis of 3 cohorts with European descent. The study found a genome-wide significant locus at chromosome 12q23.1, which mapped to *SLC25A3* (rs369920026; odds ratio [OR] for having NP, 1.68; 95% CI, 1.40-2.02; *P* = 1.30 × 10^−8^), and a suggestive variant at 13q14.2 near *CAB39L* (rs7992766; OR, 1.09; 95% CI, 1.05-1.14; *P* = 1.22 × 10^−7^). These mitochondrial phosphate carriers and calcium binding genes are expressed in brain and dorsal root ganglia. Colocalization analyses using expression quantitative loci data found that the suggestive variant was associated with expression of *CAB39L* in the brain cerebellum (*P* = 1.01 × 10^−14^). None of the previously reported variants were replicated.

**Conclusions and Relevance:**

To our knowledge, this was the largest meta-analyses of GWAS to date. It found novel genetic variants associated with NP susceptibility. These findings provide new insights into the genetic architecture of NP and important information for further studies.

## Key Points

**Question:**

Are genetic variants associated with neuropathic pain (NP) susceptibility?

**Findings:**

This genetic association study included a meta-analysis of 3 genome-wide association studies, with 4512 individuals with NP and 428 489 without, all with European descent, and identified a novel genome-wide significant locus at chromosome 12q23.1 near *SLC25A3* and a suggestive locus at chromosome 13q14.2 near *CAB39L*. These mitochondrial phosphate carriers and calcium binding genes are expressed in tissues associated with the generation of NP, including the brain and dorsal root ganglia.

**Meaning:**

These findings may provide a better understanding of genetic predisposition to NP, and this may inform the development of new treatment strategies.

## Teaser

This genetic association study identifies genetic variants associated with neuropathic pain.

## Introduction

Neuropathic pain (NP) arises as a consequence of a lesion or disease affecting the somatosensory nervous system^1^ and affects 7% to 10% of the general population.^2^ NP has heterogeneous etiologies, such as diabetes, surgery or trauma, infections such as shingles and HIV, nerve compression, nerve entrapment, and chemotherapy.^3^ It has considerable consequences for physical as well as mental health–related quality of life.^4^ Many patients do not achieve satisfactory pain relief with current drug treatments for NP.^5^ Common risk factors for NP conditions include older age, female sex, smoking, high body mass index, poor general health, and low socioeconomic status.^6,7,8^ However, these factors alone cannot fully explain the risk of developing NP. Not every patient who has an underlying relevant disease develops NP; for example, as many as 26% of individuals with diabetes were found to have NP.^9,10^ It is likely that genetic factors play a role in the risk of developing NP.^11^

A recent twins study from the United Kingdom revealed a substantial genetic contribution to NP, with a heritability estimate of 37%.^7^ Studies have shown that some rare inherited nerve pain disorders are caused by mutations in voltage-gated sodium channels (eg, *SCN9A *).^12,13^ Recently, both common and rare variants in this sodium channel were found to be associated with painful diabetic neuropathy, a common NP condition.^14,15^ Several candidate gene association studies have reported associations of genetic variants with NP.^16^ However, these studies lack consistent replication. This may be due to limited sample sizes and varying case-control definitions.^16^ Only 3 genome-wide association studies (GWASs) have been published so far, and they identified no genome-wide significant variants.^17,18,19^

The present study aimed to identify genetic variants associated with NP and to test all single nucleotide variants (SNVs) previously reported being associated with NP for replication. It was conducted as a part of the DOLORisk consortium, a multinational collaboration between research groups aiming to understand the factors associated with NP.^20^

## Methods

### Study Cohorts

Participants in this study were included from 3 independent cohorts, ie, Genetics of Diabetes Audit and Research in Tayside Scotland (GoDARTS),^21^ Generation Scotland: Scottish Family Health Study (GS:SFHS),^22^ and the United Kingdom Biobank (UKBB).^23^ GoDARTS and GS:SFHS are part of the DOLORisk consortium.^20^ GoDARTS comprises 10 149 participants with type 2 diabetes aged between 16 and 98 years recruited from Tayside, Scotland (eMethods 1 in the Supplement). GS:SFHS is a family-based population cohort of approximately 24 084 participants across Scotland aged 18 to 98 years. UKBB is a prospective biomedical resource that comprises 488 377 individuals, aged 40 to 69 years, from across the UK (eMethods 1 in the Supplement). The respective regional ethics committees approved all the study cohorts. Participants in all cohorts provided informed consent. The study is reported according to the Strengthening the Reporting of Genetic Association Studies (STREGA) reporting guideline.

### NP Phenotyping

The DOLORisk consortium developed a self-completed questionnaire, based on a recent international consensus statement on phenotyping NP (Neuropathic Pain Phenotyping by International Consensus) led by the Neuropathic Pain Special Interest Group (NeuPSIG) of the International Association for the Study of Pain,^24^ which was agreed by all participating centers.^20^ Living participants in GoDARTS (5236 with diabetes) and GS:SFHS (20 221) were recontacted by mail with the questionnaire containing: (1) chronic pain identification questions, including the presence of current pain, duration of pain, and medication intake for current pain according to the Brief Pain Inventory questionnaire^25^; (2) NP identification questions from a validated screening tool, *Douleur Neuropathique en 4 Questions*^26^ (DN4), that asks about the presence or absence of sensory symptoms; and (3) other relevant questions, as described in Hébert et al^27^ (eMethods 2 in the Supplement). All questionnaires received back from the participants (GoDARTS, 1915 [36.6%]; GS:SFHS, 7240 [35.8%]) were processed and linked to demographic data using a secure system.

Individuals with of possible NP (ie, case participants) were identified based on current reported pain and/or currently taking pain medications, pain duration of at least 3 months, and DN4 score^26^ greater than or equal to 3 of 7. Control participants were defined as those reporting no pain or not taking any pain medications at the time of completing the questionnaire. Participants who reported pain of less than 3 months’ duration or who scored less than 3 on the DN4 were excluded (eMethods 3 in the Supplement).

At the time of this study, questionnaire-based phenotyping data were not available in the UKBB. Therefore, self-reported prescribed medication linked to routine hospital admissions records were used as a proxy phenotype for NP^23^ (eMethods 3 in the Supplement). Briefly, case participants were defined as individuals with a record of the most commonly prescribed antineuropathic medicines, based on the NeuPSIG guidelines^5,28,29^ (ie, gabapentin, pregabalin, duloxetine). Control participants were those with no such reported prescriptions. Individuals reporting receipt of amitriptyline, other tricyclic antidepressants, and/or tramadol were excluded from the control and case groups, despite the potential role of these medicines in treating NP because of their frequent use to treat other conditions and consequent nonspecificity for NP. Individuals who self-reported an epilepsy diagnosis and/or any anti-epileptic medication concomitantly with a gabapentinoid alone were excluded. We calculated sensitivity and specificity of this the prescription-based phenotype by comparing with the questionnaire-based phenotype in GoDARTS (722 participants).

### Genotyping, Quality Control, and Imputation

The GoDARTS genetic data set contained genotypes for 7857 participants after quality control (QC) assessment. These were genotyped using Affymetrix version 6.0, Illumina OmniExpress BeadChips, and Illumina Infinium Broad BeadChips.^21^ Genetic data from 20 032 participants in GS:SFHS were available for analysis after QC^30^ and were genotyped on the Illumina Human OmniExpressExome-8 version 1.0 BeadChip and Illumina OmniExpressExome-8 version 1.2 BeadChip.^31^ The genome-wide genotyping for 488 377 participants in the UKBB was performed using UK Biobank Axiom and UK Biobank Lung Exome Variant Evaluation Axiom Affymetrix array.^23^ QC assessment was performed independently in all cohorts prior to and after imputation against the haplotype reference consortium release 1.1 panel (eMethods 4 in the Supplement).

### Statistical Analysis

#### Genome-wide Association Analyses and Meta-analysis

Data analysis was conducted from April 2018 to December 2019. We conducted GWAS of NP in each of these cohorts separately using a linear mixed noninfinitesimal model in BOLT-LMM version 2.3.1, which accounts for relatedness and any population stratification.^32^ The additive model was adjusted for age, as a linear variable, and sex. There was no evidence of population stratification in individual GWAS (genomic inflation factor for GoDARTS, λ = 1.001; GS:SFHS, λ = 1.008; UKBB, λ = 1.001). The meta-analysis of GWAS was conducted using a fixed-effect meta-analysis in GWAMA version 2.1 (BMC Bioinformatics) (eMethods 5 in the Supplement).^33^ We combined the summary statistics from GoDARTS and GS:SFHS in stage 1 and UKBB in stage 2. We performed a sensitivity analysis by combining GS:SFHS and UKBB GWAS as well as GoDARTS and UKBB GWAS separately. Stratified analysis was performed based on diabetes status in UKBB and was not performed in GS:SFHS because of a sample size (44 participants with diabetes).

#### In Silico Functional Annotation, Colocalization, and Lookups for Pain-Related Traits

Functional annotation was performed using the University of California Santa Cruz Genome Browser resource,^34^ RegulomeDB,^35^ HaploReg^36^ version 1, and FUMA.^37^ We performed fine mapping method implemented in FINEMAP^38^ version 1.1 using summary statistics data (*z* scores) that comprise a 500 kilobase region centered on the lead SNV from stage 2. Expression quantitative trait loci (eQTL) analysis was performed using the genotype-tissue expression (GTEx)^39^ version 7, eQTL database of human dorsal root ganglia (DRG),^40^ and brain xQTL serve.^41^ Colocalization analysis was performed using coloc package version 5.1.0 in R version 3.6.3 (R Project for Statistical Computing).^42^ We conducted lookups for the association of lead SNVs with pain-related traits in GeneATLAS^43^ (eMethods 6 in the Supplement).

#### SNV-Based Heritability

We used summary statistics data from the meta-analysis to estimate the SNV-based heritability in a liability scale. Linkage Disequilibrium Score Regression (LDSC) software^44^ was used for this process (eMethods 7 in the Supplement).

#### Replication of SNVs Previously Associated With NP

A total of 51 SNVs previously found to be associated with the presence of NP from a published systematic review^16^ were tested in all 3 cohorts and the meta-analysis (stage 1 and stage 2). SNVs associated with nominal significance with an unadjusted *P* < .05 from stage1 and stage 2 were selected for combined analysis with the prior studies. SNVs were considered replicated if they were significantly associated after Bonferroni correction adjustment for multiple comparisons (*P* < .0009; α = .05/51 variants) and consistent with the direction of association reported in original publication. SNVs with *P* > 5 × 10^−8^ and *P* < 5 × 10^−5^ were considered suggestive SNVs, while those with *P* < 5 × 10^−8^ were considered genome-wide significant.

## Results

### Study Design

**Figure 1.**
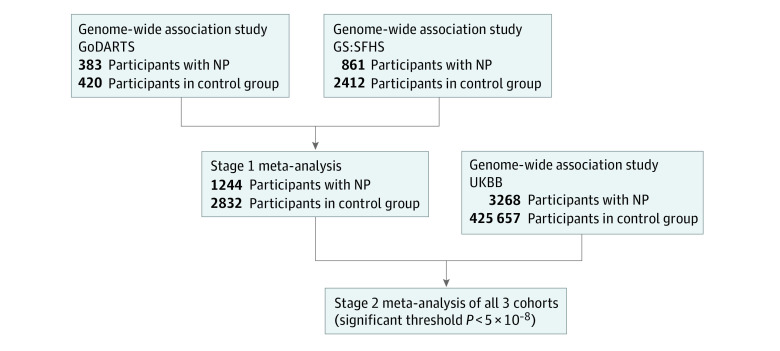
Study Design The study included meta-analysis of 3 independent genome-wide association studies for neuropathic pain (NP) using case and control participants from Genetics of Diabetes Audit and Research in Tayside, Scotland (GoDARTS), Generation Scotland: Scottish Family Health Study (GS:SFHS), and the United Kingdom BioBank (UKBB). Self-reported questionnaires were used to define NP in stage 1, followed by meta-analysis of genome-wide association studies of NP using all 3 cohorts in stage 2.

We identified 482 NP cases of 1597 participants (30.5%; 222 [46.1%] women; mean age [SD] age, 69.5 [10.2] years) in GoDARTS (where all participants have diabetes) and 932 NP cases of 7240 participants (12.87%; 624 [67.0%] women; mean [SD] age, 59 [11.1] years) in the GS:SFHS based on the agreed phenotype, broadly consistent with prevalence rates in published studies.^2^ We identified 560 participants (29.2%; 173 [30.9%] women; mean [SD] age, 71 [9.1] years) and 2642 participants (36.5%; 1532 [58.0%] women; mean [SD] age, 58 [13.4] years) for the control group in GoDARTS and GS:SFHS, respectively. In the UKBB, we identified 3268 NP cases (1949 [59.6%] women; mean [SD] age, 56.2 [11.1] years) and 425 657 controls (226 568 [53.2%] women; mean [SD] age, 57.5 [12.1] years) of European descent using the prescribing-based phenotyping, which showed high specificity (89.0%) and slightly lower sensitivity (80.3%) against the questionnaire-based phenotype (eTable 1 in the Supplement). We conducted the meta-analysis of GWAS in 2 stages because of differences in the phenotyping methods (Figure 1). In stage 1, a total of 1244 case participants (713 [57.3%] women; mean [SD] age, 64.4 [10.6] years) and 2832 control participants (1249 [44.1%] women; mean [SD] age, 64.8 [11.2] years) had genetic data available and were used for the meta-analysis of GWAS from the GoDARTS and GS:SFHS cohorts, as these used consistent phenotyping. In stage 2, we combined summary GWAS results from all 3 cohorts (GoDARTS, GS:SFHS, and UKBB) to maximize the study power, with a total of 4512 NP case participants (2662 [58.9%] women; mean [SD] age, 61.7 [10.8] years) and 428 489 control participants (227 817 [53.2%] women; mean age, 62.3 [11.5] years).

### Meta-analyses

**Figure 2.**
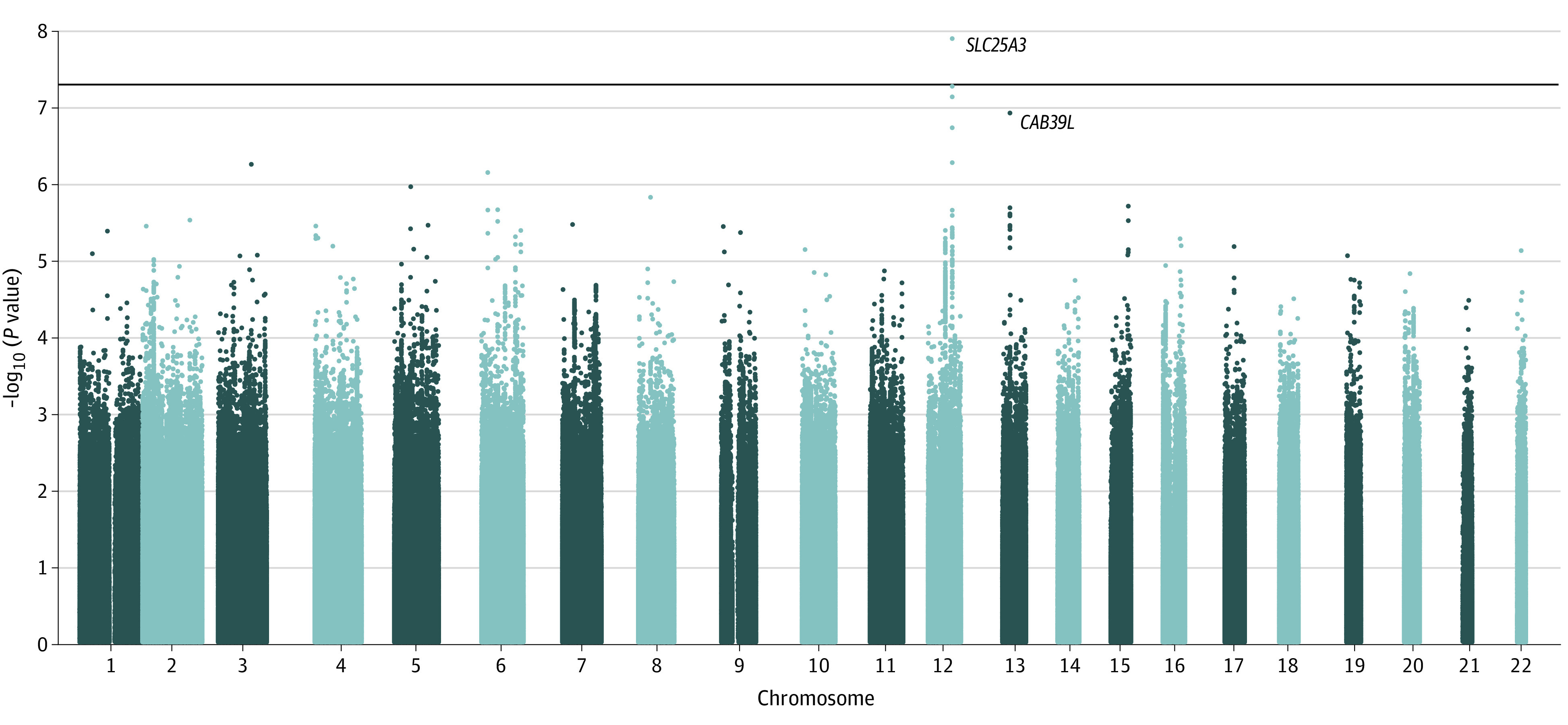
Manhattan Plot Showing Single Nucleotide Variant Associations in Stage 2 Meta-analysis of Genome-wide Association Studies The horizontal line indicates the genome-wide significance threshold (*P* < 5 × 10^−8^). The x-axis represents physical position along with the 22 autosomal chromosomes; y-axis represents negative logarithm of association *P* value. Each dot on the plot represents millions of imputed single nucleotide variant across the whole genome. The most significant single nucleotide variants mapped to nearby genes are labeled.

**Figure 3.**
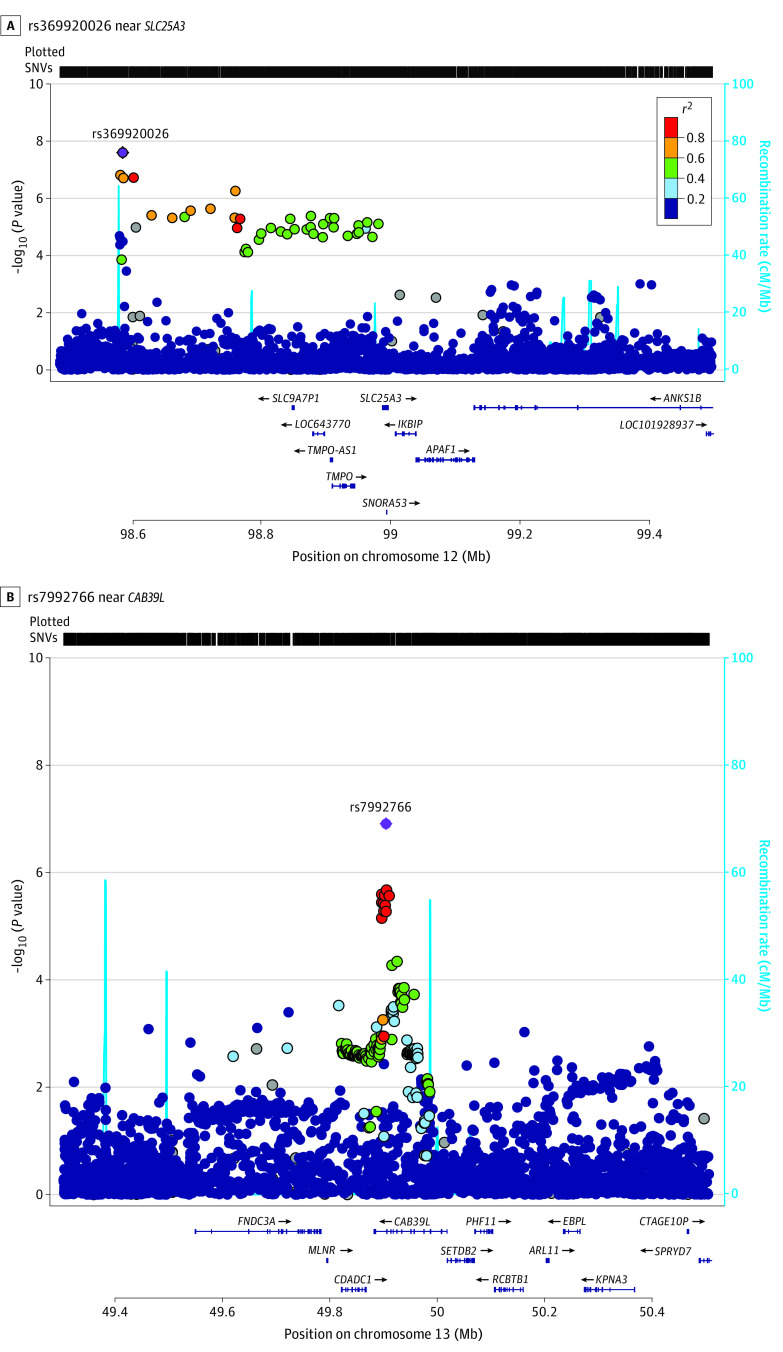
Regional Association Plots of the Most Significant Single Nucleotide Variants (SNVs) in the Stage 2 Meta-Analysis

**Figure 4.**
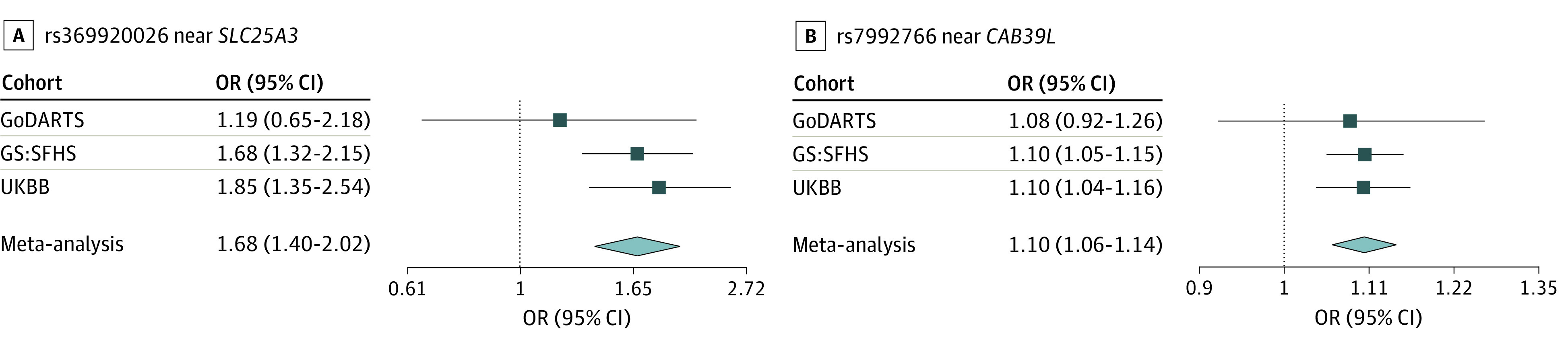
Forest Plot for the Most Significant Single Nucleotide Variants in the Stage 2 Meta-analysis GoDARTS indicates Genetics of Diabetes Audit and Research in Tayside Scotland; GS:SFHS, Generation Scotland: Scottish Family Health Study; and UKBB, United Kingdom BioBank.

The meta-analysis of all 3 cohorts yielded a novel genome-wide significant variant, rs369920026, at 12q23.1 associated with NP (odds ratio [OR], 1.68; 95% CI, 1.40-2.02; *P* = 1.30 × 10^−8^) with no heterogeneity (Table, Figure 2, Figure 3, and Figure 4). The quantile-quantile plot showed no evidence of genomic inflation (λ = 1.041) (eFigure 1 in the Supplement). Suggestive SNVs at this locus (rs185663675, *P* = 5.46 × 10^−8^; rs17027891, *P* = 7.50 × 10^−8^; rs17027910, 1.90 × 10^−7^) were in linkage disequilibrium (LD) with the lead SNV (*r*^2^ > 0.6; D′ = 1) (eTable 2 in the Supplement). All these variants have relatively low frequencies (minor allele frequency [MAF] ≤0.008) and were well imputed with quality scores between 0.967 and 0.994. We found that directly typed variant (rs12309615 at 12q23.1) was associated with NP, with an OR of 1.27 (95% CI, 1.13-1.41; *P* = 2.7 × 10^−5^; MAF, 0.01; *I*^2^ = 0.04; *r*^2^ = 0.4; D′ = 1) and was genotyped with a high-quality call rate (eFigure 2 in the Supplement). In sensitivity analysis, the lead SNV remained significant at the genome-wide level (rs369920026) in the combined GWAS from GS:SFHS and UKBB and achieved significance with and OR of 1.28 (95% CI, 1.11-1.48; *P* = 1.63 × 10^−4^) in the combined analysis of GoDARTS and UKBB (eTable 3 in the Supplement).

**Table.** Summary Statistics of the Most Significant SNVs From Stage 1 and Stage 2 Meta-analysis

SNV	Chromosome	Base position^a^	EA/NEA	EAF	Cohort	OR (95% CI)	*P* value	*I* ^2^	Gene
rs369920026	12	98585582	A/G	0.006	GoDARTS	1.19 (0.65-2.19)	.57	NA	*SLC25A3*
					GS:SFHS	1.68 (1.32-2.15)	2.10 × 10^−5^	NA	
					Stage 1^b^	1.61 (1.28-2.02)	1.73 × 10^−5^	0.01	
					UKBB	1.85 (1.35-2.54)	1.29 × 10^−4^	NA	
					Stage 2^c^	1.68 (1.40-2.02)	1.30 × 10^−8^	0.00	
rs7992766	13	49905672	A/C	0.750	GoDARTS	1.08 (0.93-1.27)	.10	NA	*CAB39L*
					GS:SFHS	1.10 (1.05-1.15)	3.2 × 10^−5^	0.03	
					Stage 1^b^	1.10 (1.05-1.15)	2.41 × 10^−5^	0.23	
					UKBB	1.10 (1.04-1.16)	9.00 × 10^−4^	NA	
					Stage 2^c^	1.09 (1.05-1.14)	1.22 × 10^−7^	0.31	
rs112990863	3	88714964	T/A	0.007	GoDARTS	1.10 (0.63-1.93)	.68	NA	*EPHA3*
					GS:SFHS	1.85 (1.50-2.28)	8.80 × 10^−9^	NA	
					Stage 1^b^	1.74 (1.43-2.11)	3.73 × 10^−8^	0.35	
					UKBB	1.01 (0.76-1.34)	.96	NA	
					Stage 2^c^	1.46 (1.24-1.72)	8.99 × 10^−6^	0.85	

Abbreviations: EA, effect allele; EAF, effect allele frequency; GoDARTS, Genetics of Diabetes Audit and Research in Tayside Scotland; GS:SFHS, Generation Scotland: Scottish Family Health Study; NA, not applicable; NEA, noneffect allele; OR, odds ratio; SNV, single nucleotide variant; UKBB, United Kingdom Biobank.

^a^ Base position based on National Center for Biotechnology Information build 37.

^b^ Stage 1 was a meta-analysis of GoDARTS and GS:SFHS.

^c^ Stage 2 was an overall meta-analysis of GoDARTS, GS:SFHS, and UKBB.

The lead SNV (rs369920026) falls in an intronic region of long noncoding RNA (lncRNA), *AC016152**.1*, and the nearest gene is within 400 kilobase of the transcription start site (TSS) of solute carrier family 25 member 3 (*SLC25A3 *[OMIM 600370]). Data from RegulomeDB indicated that rs185663675 is located within a DNase I hypersensitivity site in brain tissues, lies in an open chromatin state in brain tissues (chromHMM score, <8),^37^ and disrupts a signal transducer and activator of transcription (STAT) motif; rs17027910 had a very high RegulomeDB probability score of 0.90 of 1, indicating that it is likely to be involved in regulatory effects. We used FINEMAP to identify associated variants in the 12q23.1 region (500 kilobase) centered on rs369920026 with 1808 SNVs from stage 2, allowing for at most 2 associated variants. It indicated that rs369920026 had the highest probability of being an associated variant, with a posterior inclusion probability (PIP) of 0.63. *SLC25A3* is highly expressed in brain and DRG tissues; however, the eQTL information for these SNVs (MAF <1%) are not available in GTEx version 7. There are 3 other genes in the vicinity of the association on chromosome 12q23.1, including solute carrier family 9 members 7 pseudogene 1 (*SLC9A7P1*), thymopoietin (*TMPO *[OMIM 188380]), and inhibitor of nuclear factor kappa-B kinase-interacting protein (*IKBIP *[OMIM 609861]). However, none of these are expressed in brain and DRG tissues. Lookups in the GeneATLAS^43^ indicated that the lead SNV was also associated with intervertebral disc problems and fibromyalgia in the UKBB cohort (eTable 4 in the Supplement).

We also found a suggestive locus at 13q14.2 (rs7992766; OR, 1.09; 95% CI, 1.05-1.14; *P* = 1.22 × 10^−7^; *I*^2^ = 0.2; MAF, 0.25), which lies within the intronic region of calcium-binding protein 39-like (*CAB39L *[OMIM 612175]) and overlaps with DNase hypersensitivity in brain tissues. It is significantly associated with mRNA expression of *CAB39L* in musculoskeletal tissue (*P* = 9.40 × 10^−25^), brain cerebellum (*P* = 1.01 × 10^−14^), and brain cortex (*P* = 7.6 × 10^−7^) (eFigure 3 in the Supplement). *CAB39L* had a posterior probability (PP4 >0.1) for colocalization for NP and brain cerebellum tissue eQTLs. Furthermore, rs7334929 (cis-eQTL) tag SNV was significantly associated with the expression of *CAB39L* in the DRG^40^ (*P* = 8.09 × 10^−7^) (eTable 5 in the Supplement). It was also associated with lower limb ulcers and neck/shoulder pain in the UKBB (*P* = .001) (eTable 4 in the Supplement).

The most significant variant, rs112990863, at 3p11.1 near ephrin receptor tyrosine kinase A3 (*EPHA3 *[OMIM 179611]) from stage 1 (OR, 1.29; 95% CI, 1.16-1.14; *P* = 3.73 × 10^−8^; MAF, 0.007; λ = 1.023) (eFigure 4, eFigure 5, and eTable 6 in the Supplement) dropped to suggestive significance only (OR, 1.46; 95% CI, 1.24-1.72; *P* = 8.99 × 10^−6^) with high heterogeneity (*I*^2^ = 0.85) in stage 2 (Table). In the stratified analysis, this SNV did not achieve significant association with NP that included UKBB participants with diabetes (425 case participants; 17 435 control participants) and without diabetes (2843 case participants; 408 132 control participants).

SNV-based heritability was estimated using the LDSC^44^ and full GWAS summary results from stage 1 (33% [SE, 0.14%]) and stage 2 (20% [SE, 0.11%]) meta-analysis. Association results of previously reported variants^16^ in stage 1 meta-analysis, UKBB, and stage 2 meta-analysis are presented in eTable 7 in the Supplement. Only one variant (rs1901531) in Beta-2-Microglobulin^45^ (*B2M *[OMIM 109700]) was found to be significantly associated in stage 1 (unadjusted *P* = .02), stage 2 (unadjusted *P* = .04), and combined analysis (*P* = .03) with prior results (eTable 8 in the Supplement). However, this variant was not significant when we applied multiple corrections either in independent cohorts or meta-analysis.

## Discussion

To our knowledge, this is the largest meta-analysis of GWAS of NP published to date, and it has identified a novel genome-wide significant locus at 12q23.1 and a suggestive locus at 13q14.2. The minor allele (MA) of the most significant variant (rs369920026) in stage 2 at 12q23.1 conferred risk of having NP, and the frequency was 0.006 in European populations. The suggestive SNVs (rs185663675, rs1702789, and rs17027910) at this locus are in LD with the lead variant and were imputed with high quality score of 0.99. The directly typed variant (rs12309615; MAF, 0.01) at this locus was genotyped with good quality and significantly associated with NP, which could drive these imputed SNVs. Our fine mapping of the region (12q23.1) indicated that the lead SNV could be a candidate causal variant. In stage 1, rs112990863 at 3p11.1 was found to be genome-wide significant, but the association became weak in stage 2 with high heterogeneity. Due to the so-called winner’s curse,^46^ the effect size of this SNV is likely to be overestimated in the GS:SFHS GWAS. The SNV-heritability analyses found that NP has a moderate heritability (20%) which is slightly higher than the estimated heritability (10%-12%) for multisite chronic pain from a recent large GWAS.^47,48^ It is lower than the heritability estimated by a previous twins study using the outcome of chronic widespread pain with neuropathic characteristics (37%).^7^ Notably, the previously reported genetic variants failed to replicate in this study after multiple corrections. The reason for this may be that the original studies found relatively weak associations and were investigating the associations in small sample sizes, resulting in false-positive findings; the present study had a larger sample size than any of the previous studies that we are aware of.^16^ Differences in the phenotypes could be an explanation for some of the variations. Future research is required to examine this.

The lead variants are within introns of the lncRNA *AC016152**.1* and the closest coding gene (within 400 kilobase of the TSS) is *SLC25A3*. *SLC25A3* encodes a mitochondrial phosphate carrier protein in humans that plays a role in oxidative phosphorylation,^49^ cytochrome c oxidase biogenesis,^50^ and calcium ion homeostasis.^51^ It is highly expressed in the brain, DRG, heart, and skeletal muscle. A variant in *SLC25A3* has been reported to indirectly regulate the mitochondrial permeability transition pore in response to calcium.^52^ Studies using animal models have suggested that mitochondrial dysfunction plays a role in the pathogenesis of NP in the context of traumatic, chemotherapy-related, and diabetic peripheral neuropathy.^53,54,55^ Moreover, a recent gene expression study in a diabetic peripheral neuropathy animal model (streptozotocin-induced diabetic DBA/2J mice) reported an association of downregulation of *SLC25A3* in sciatic nerve with type 1 diabetes.^56^ To date, there are no data available to show whether the lead variant is associated with the expression of *SLC25A3* gene in the brain or relevant tissues, to our knowledge. However, data from pain networks database indicate a potential indirect interaction between 6 pain-related genes (*NOS2*, *CDK5*, *TNFRSF1A*, *TNFRSF1B*, *ADRBK1*, and *ADRB2*) and *SLC25A3*.^57^ This database also shows that *SLC25A3* mRNA expression is reduced in DRG following traumatic nerve injury models in the mouse and rat. Adult mouse proteome data indicate that *SLC25A3* is significantly downregulated in DRG after NP induction.^58^ The differential expression of *SLC25A3* in experimental models of NP is suggestive of a role in NP pathogenesis; however, mechanistic studies to determine the impact of this gene on NP-related behavior and excitability will ultimately be needed. Moreover, the most significant SNV could affect the expression of other nearby genes and may have indirect regulatory effects, as they overlap with STAT/STAT3 binding sites and DNase I hypersensitivity site in brain tissues. A recent animal model study reported the role of STAT3 in NP development.^59^

The suggestive locus at 13q14.2 falls in the intronic region of *CAB39L*. *CAB39L* encodes a calcium-binding protein 39-like, which is known to be involved in apoptosis,^60^ and mammalian target of rapamycin signaling pathway, which acts as an important regulator of pain processing.^61^ It is significantly expressed in the tissues relevant to NP generation, which include DRG^40^ and the brain cortex.^41^ Furthermore, colocalization analysis indicated that this suggestive variant was associated with the expression of *CAB39L* significantly in the brain cerebellum.

We also found a weak association of the genome-wide significant variant with pain-related traits including disc problems and fibromyalgia and the suggestive variant with lower limb ulcer (a consequence of diabetic neuropathy) and neck/shoulder pain in the UKBB.^43^ These associations are interesting but need to be confirmed in independent data sets. Future studies with enough power and similar phenotypes are needed to validate our findings. More investigations on the functional effects of these genes are required to ascertain their role in NP.

### Limitations

This study has some limitations. The cohorts (GoDARTS and GS:SFHS/UKBB) were dissimilar in that the former comprises only people with diabetes, while the latter are general population samples. However, our sensitivity analysis, including only GS:SFHS and UKBB or GoDARTS and UKBB, showed that the lead SNVs remained significant, with consistent effect sizes. While there may have been a phenotypic bias in response rates to specific questionnaires such as DN4,^27^ there is no reason to suppose that genetic factors contributed to nonresponse. This potential bias is therefore unlikely to affect the overall results. NP case and control definitions were not identical between stage 1 (GoDARTS and GS:SFHS) and the UKBB. Moreover, the UKBB control group may include participants with other chronic pain (non-NP) and those with no pain. These differences may introduce phenotypic heterogeneity to the analyses and are likely to reduce the identification of genetic signals associated with the traits. However, the prescribing-based phenotyping had a high specificity and reasonable sensitivity.

## Conclusions

To our knowledge, this is the largest meta-analysis of GWAS of NP to date. It found novel risk loci near *SLC25A3* and *CAB39L* that are expressed in tissues associated with the generation of NP, including the brain and DRG. These merit further investigation. Our findings provide a basis for better understanding of the genetic predisposition to NP.

## Notes

Supplement. **eMethods 1.** Description of Study Cohorts **eMethods 2.** Self-completed Questionnaire Data **eMethods 3.** NP Phenotyping **eMethods 4.** Genotyping, Quality Control, and Imputation **eMethods 5.** Genome-Wide Association Analyses and Meta-analyses **eMethods 6.** In Silico Functional Annotation, Expression Quantitative Loci, and Colocalization Analysis **eMethods 7.** SNV-Based Heritability
eReferences.
**eTable 1.** Sensitivity and Specificity of Neuropathic Pain Phenotyping Methods in GoDARTS **eTable 2.** Most Significant SNVs (*P* < 5 × 10^-5^) Associated With NP in the Meta-analysis of All 3 Cohorts (GoDARTS, GS:SFHS, and UKBB) **eTable 3.** Sensitivity Analysis of the Most Significant SNVs Associated With NP in the Stage 2 Meta-analysis **eTable 4.** The Most Significant SNVs Associated With NP From the Overall Meta-analysis and Related Traits **eTable 5.** Expression Quantitative Trait Loci Information for the Most Signficant Genetic Loci Associated With NP in Human Dorsal Root Ganglia (eQTL DRG) and Brain Cortex (brain xQTL serve) **eTable 6. **Most Significant SNVs (*P* < 5 × 10^-5^) Associated With NP in Stage 1 (GoDARTS and GS:SFHS) and UKBB Study **eTable 7.** Association Results of Previously Reported SNVs in Each Study and Meta-analysis (Stage 1 and Stage 2) **eTable 8.** Combined Analysis of the Present Study Summary Statistics (Stage 1 and Stage 2) and the Original Study Summary Statistics **eFigure 1.** Quantile-Quantile Plot for the Results of Stage 2 Meta-analysis (GoDARTS, GS:SFHS, and UKBB) GWAS **eFigure 2.** Scattershot of Directly Typed Variant at Chromosome 12q23.1 From the UKBB Data **eFigure 3.** Multitissue eQTL Comparison for a Promising Candidate SNV for NP and Correlation With Expression of *CAB39L* **eFigure 4.** Plots Showing the P Value of Association Tests for SNVs With Possible NP in Stage 1 Meta-analysis (GoDARTS and GS:SFHS) **eFigure 5.** Regional Association Plot of an Index SNV in Stage 1 Meta-analysis (GoDARTS and GS:SFHS)

## References

[1] Treede RD, Jensen TS, Campbell JN, . Neuropathic pain: redefinition and a grading system for clinical and research purposes. Neurology. 2008;70(18):1630-1635. doi:10.1212/01.wnl.0000282763.29778.59 18003941

[2] van Hecke O, Austin SK, Khan RA, Smith BH, Torrance N. Neuropathic pain in the general population: a systematic review of epidemiological studies. Pain. 2014;155(4):654-662. doi:10.1016/j.pain.2013.11.013 24291734

[3] Colloca L, Ludman T, Bouhassira D, . Neuropathic pain. Nat Rev Dis Primers. 2017;3:17002. doi:10.1038/nrdp.2017.2 28205574 PMC5371025

[4] Jensen MP, Chodroff MJ, Dworkin RH. The impact of neuropathic pain on health-related quality of life: review and implications. Neurology. 2007;68(15):1178-1182. doi:10.1212/01.wnl.0000259085.61898.9e 17420400

[5] Finnerup NB, Attal N, Haroutounian S, . Pharmacotherapy for neuropathic pain in adults: a systematic review and meta-analysis. Lancet Neurol. 2015;14(2):162-173. doi:10.1016/S1474-4422(14)70251-0 25575710 PMC4493167

[6] VanDenKerkhof EG, Mann EG, Torrance N, Smith BH, Johnson A, Gilron I. An epidemiological study of neuropathic pain: symptoms in Canadian adults. Pain Res Manag. 2016;2016:9815750. doi:10.1155/2016/9815750 27445636 PMC4904601

[7] Momi SK, Fabiane SM, Lachance G, Livshits G, Williams FMK. Neuropathic pain as part of chronic widespread pain: environmental and genetic influences. Pain. 2015;156(10):2100-2106. doi:10.1097/j.pain.0000000000000277 26121255 PMC4770357

[8] Bouhassira D, Lantéri-Minet M, Attal N, Laurent B, Touboul C. Prevalence of chronic pain with neuropathic characteristics in the general population. Pain. 2008;136(3):380-387. doi:10.1016/j.pain.2007.08.013 17888574

[9] Abbott CA, Malik RA, van Ross ERE, Kulkarni J, Boulton AJM. Prevalence and characteristics of painful diabetic neuropathy in a large community-based diabetic population in the U.K. Diabetes Care. 2011;34(10):2220-2224. doi:10.2337/dc11-1108 21852677 PMC3177727

[10] Davies M, Brophy S, Williams R, Taylor A. The prevalence, severity, and impact of painful diabetic peripheral neuropathy in type 2 diabetes. Diabetes Care. 2006;29(7):1518-1522. doi:10.2337/dc05-2228 16801572

[11] Calvo M, Davies AJ, Hébert HL, . The genetics of neuropathic pain from model organisms to clinical application. Neuron. 2019;104(4):637-653. doi:10.1016/j.neuron.2019.09.018 31751545 PMC6868508

[12] Yang Y, Wang Y, Li S, . Mutations in *SCN9A*, encoding a sodium channel alpha subunit, in patients with primary erythermalgia. J Med Genet. 2004;41(3):171-174. doi:10.1136/jmg.2003.012153 14985375 PMC1735695

[13] Fertleman CR, Baker MD, Parker KA, . *SCN9A* mutations in paroxysmal extreme pain disorder: allelic variants underlie distinct channel defects and phenotypes. Neuron. 2006;52(5):767-774. doi:10.1016/j.neuron.2006.10.006 17145499

[14] Blesneac I, Themistocleous AC, Fratter C, . Rare NaV1.7 variants associated with painful diabetic peripheral neuropathy. Pain. 2018;159(3):469-480. doi:10.1097/j.pain.0000000000001116 29176367 PMC5828379

[15] Li QS, Cheng P, Favis R, Wickenden A, Romano G, Wang H. *SCN9A* variants may be implicated in neuropathic pain associated with diabetic peripheral neuropathy and pain severity. Clin J Pain. 2015;31(11):976-982. doi:10.1097/AJP.0000000000000205 25585270 PMC4894774

[16] Veluchamy A, Hébert HL, Meng W, Palmer CNA, Smith BH. Systematic review and meta-analysis of genetic risk factors for neuropathic pain. Pain. 2018;159(5):825-848. doi:10.1097/j.pain.0000000000001164 29351172

[17] Meng W, Deshmukh HA, van Zuydam NR, ; Wellcome Trust Case Control Consortium 2 (WTCCC2); Surrogate Markers for Micro- and Macro-Vascular Hard Endpoints for Innovative Diabetes Tools (SUMMIT) Study Group. A genome-wide association study suggests an association of Chr8p21.3 (*GFRA2*) with diabetic neuropathic pain. Eur J Pain. 2015;19(3):392-399. doi:10.1002/ejp.560 24974787 PMC4737240

[18] Warner SC, van Meurs JB, Schiphof D, . Genome-wide association scan of neuropathic pain symptoms post total joint replacement highlights a variant in the protein-kinase C gene. Eur J Hum Genet. 2017;25(4):446-451. doi:10.1038/ejhg.2016.196 28051079 PMC5386416

[19] Meng W, Deshmukh HA, Donnelly LA, ; Wellcome Trust Case Control Consortium 2 (WTCCC2); Surrogate Markers for Micro- and Macro-Vascular Hard Endpoints for Innovative Diabetes Tools (SUMMIT) study group. A genome-wide association study provides evidence of sex-specific involvement of Chr1p35.1 (*ZSCAN20-TLR12P*) and Chr8p23.1 (*HMGB1P46*) with diabetic neuropathic pain. EBioMedicine. 2015;2(10):1386-1393. doi:10.1016/j.ebiom.2015.08.001 26629533 PMC4634194

[20] Pascal MMV, Themistocleous AC, Baron R, . DOLORisk: study protocol for a multi-centre observational study to understand the risk factors and determinants of neuropathic pain. Wellcome Open Res. 2019;3:63. doi:10.12688/wellcomeopenres.14576.2 30756091 PMC6364377

[21] Hébert HL, Shepherd B, Milburn K, . Cohort profile: Genetics of Diabetes Audit and Research in Tayside Scotland (GoDARTS). Int J Epidemiol. 2018;47(2):380-381j. doi:10.1093/ije/dyx140 29025058 PMC5913637

[22] Smith BH, Campbell A, Linksted P, . Cohort profile: Generation Scotland: Scottish Family Health Study (GS:SFHS): the study, its participants and their potential for genetic research on health and illness. Int J Epidemiol. 2013;42(3):689-700. doi:10.1093/ije/dys084 22786799

[23] Bycroft C, Freeman C, Petkova D, . The UK Biobank resource with deep phenotyping and genomic data. Nature. 2018;562(7726):203-209. doi:10.1038/s41586-018-0579-z 30305743 PMC6786975

[24] van Hecke O, Kamerman PR, Attal N, . Neuropathic pain phenotyping by international consensus (NeuroPPIC) for genetic studies: a NeuPSIG systematic review, Delphi survey, and expert panel recommendations. Pain. 2015;156(11):2337-2353. doi:10.1097/j.pain.0000000000000335 26469320 PMC4747983

[25] Cleeland CS, Ryan KM. Pain assessment: global use of the Brief Pain Inventory. Ann Acad Med Singap. 1994;23(2):129-138. 8080219

[26] Attal N, Lanteri-Minet M, Laurent B, Fermanian J, Bouhassira D. The specific disease burden of neuropathic pain: results of a French nationwide survey. Pain. 2011;152(12):2836-2843. doi:10.1016/j.pain.2011.09.014 22019149

[27] Hébert HL, Veluchamy A, Baskozos G, . Cohort profile: DOLORisk Dundee: a longitudinal study of chronic neuropathic pain. BMJ Open. 2021;11(5):e042887. doi:10.1136/bmjopen-2020-042887 33952538 PMC8103377

[28] Cruccu G, Sommer C, Anand P, . EFNS guidelines on neuropathic pain assessment: revised 2009. Eur J Neurol. 2010;17(8):1010-1018. doi:10.1111/j.1468-1331.2010.02969.x 20298428

[29] National Institute for Health and Care Excellence. Neuropathic pain—the pharmacological management of neuropathic pain in adults in non-specialist. Updated September 22, 2020. Accessed November 1, 2021. https://www.nice.org.uk/guidance/cg173

[30] Nagy R, Boutin TS, Marten J, . Exploration of haplotype research consortium imputation for genome-wide association studies in 20,032 Generation Scotland participants. Genome Med. 2017;9(1):23. doi:10.1186/s13073-017-0414-4 28270201 PMC5339960

[31] Kerr SM, Campbell A, Marten J, . Electronic health record and genome-wide genetic data in Generation Scotland participants. Wellcome Open Res. 2017;2(May):85. doi:10.12688/wellcomeopenres.12600.1 29062915 PMC5645708

[32] Loh PR, Tucker G, Bulik-Sullivan BK, . Efficient bayesian mixed-model analysis increases association power in large cohorts. Nat Genet. 2015;47(3):284-290. doi:10.1038/ng.3190 25642633 PMC4342297

[33] Mägi R, Morris AP. GWAMA: software for genome-wide association meta-analysis. BMC Bioinformatics. 2010;11(ii):288. doi:10.1186/1471-2105-11-288 20509871 PMC2893603

[34] Speir ML, Zweig AS, Rosenbloom KR, . The UCSC Genome Browser database: 2016 update. Nucleic Acids Res. 2016;44(D1):D717-D725. doi:10.1093/nar/gkv1275 26590259 PMC4702902

[35] Boyle AP, Hong EL, Hariharan M, . Annotation of functional variation in personal genomes using RegulomeDB. Genome Res. 2012;22(9):1790-1797. doi:10.1101/gr.137323.112 22955989 PMC3431494

[36] Ward LD, Kellis M. HaploReg: a resource for exploring chromatin states, conservation, and regulatory motif alterations within sets of genetically linked variants. Nucleic Acids Res. 2012;40(Database issue):D930-D934. doi:10.1093/nar/gkr917 22064851 PMC3245002

[37] Watanabe K, Taskesen E, van Bochoven A, Posthuma D. Functional mapping and annotation of genetic associations with FUMA. Nat Commun. 2017;8(1):1826. doi:10.1038/s41467-017-01261-5 29184056 PMC5705698

[38] Benner C, Spencer CCA, Havulinna AS, Salomaa V, Ripatti S, Pirinen M. FINEMAP: efficient variable selection using summary data from genome-wide association studies. Bioinformatics. 2016;32(10):1493-1501. doi:10.1093/bioinformatics/btw018 26773131 PMC4866522

[39] Battle A, Brown CD, Engelhardt BE, Montgomery SB; GTEx Consortium; Laboratory, Data Analysis & Coordinating Center (LDACC)—Analysis Working Group; Statistical Methods Groups—Analysis Working Group; Enhancing GTEx (eGTEx) Groups; NIH Common Fund; NIH/NCI; NIH/NHGRI; NIH/NIMH; NIH/NIDA; Biospecimen Collection Source Site—NDRI; Biospecimen Collection Source Site—RPCI; Biospecimen Core Resource—VARI; Brain Bank Repository—University of Miami Brain Endowment Bank; Leidos Biomedical—Project Management; ELSI Study; Genome Browser Data Integration &Visualization—EBI; Genome Browser Data Integration &Visualization—UCSC Genomics Institute, University of California Santa Cruz; Lead analysts; Laboratory, Data Analysis &Coordinating Center (LDACC); NIH program management; Biospecimen collection; Pathology; eQTL manuscript working group. Genetic effects on gene expression across human tissues. Nature. 2017;550(7675):204-213. doi:10.1038/nature24277 29022597 PMC5776756

[40] Parisien M, Khoury S, Chabot-Doré AJ, . Effect of human genetic variability on gene expression in dorsal root ganglia and association with pain phenotypes. Cell Rep. 2017;19(9):1940-1952. doi:10.1016/j.celrep.2017.05.018 28564610 PMC5524461

[41] Ng B, White CC, Klein HU, . An xQTL map integrates the genetic architecture of the human brain’s transcriptome and epigenome. Nat Neurosci. 2017;20(10):1418-1426. doi:10.1038/nn.4632 28869584 PMC5785926

[42] Giambartolomei C, Vukcevic D, Schadt EE, . Bayesian test for colocalisation between pairs of genetic association studies using summary statistics. PLoS Genet. 2014;10(5):e1004383. doi:10.1371/journal.pgen.1004383 24830394 PMC4022491

[43] Canela-Xandri O, Rawlik K, Tenesa A. An atlas of genetic associations in UK Biobank. Nat Genet. 2018;50(11):1593-1599. doi:10.1038/s41588-018-0248-z 30349118 PMC6707814

[44] Speed D, Balding DJ. Better estimation of SNP heritability from summary statistics provides a new understanding of the genetic architecture of complex traits. bioRxiv. Preprint posted online March 19, 2018. doi:10.1101/284976

[45] Kallianpur AR, Jia P, Ellis RJ, ; CHARTER Study Group. Genetic variation in iron metabolism is associated with neuropathic pain and pain severity in HIV-infected patients on antiretroviral therapy. PLoS One. 2014;9(8):e103123. doi:10.1371/journal.pone.0103123 25144566 PMC4140681

[46] Palmer C, Pe’er I. Statistical correction of the winner’s curse explains replication variability in quantitative trait genome-wide association studies. PLoS Genet. 2017;13(7):e1006916. doi:10.1371/journal.pgen.1006916 28715421 PMC5536394

[47] Johnston KJA, Ward J, Ray PR, . Sex-stratified genome-wide association study of multisite chronic pain in UK Biobank. PLoS Genet. 2021;17(4):e1009428. doi:10.1371/journal.pgen.1009428 33830993 PMC8031124

[48] Johnston KJA, Adams MJ, Nicholl BI, . Genome-wide association study of multisite chronic pain in UK biobank. Genome-wide Assoc study multisite chronic pain UK Biobank. bioRxiv. Preprint posted online December 20, 2018. doi:10.1101/502807

[49] Mayr JA, Merkel O, Kohlwein SD, . Mitochondrial phosphate-carrier deficiency: a novel disorder of oxidative phosphorylation. Am J Hum Genet. 2007;80(3):478-484. doi:10.1086/511788 17273968 PMC1821108

[50] Boulet A, Vest KE, Maynard MK, . The mammalian phosphate carrier *SLC25A3* is a mitochondrial copper transporter required for cytochrome *c* oxidase biogenesis. J Biol Chem. 2018;293(6):1887-1896. doi:10.1074/jbc.RA117.000265 29237729 PMC5808751

[51] Shimoyama M, De Pons J, Hayman GT, . The Rat Genome Database 2015: genomic, phenotypic and environmental variations and disease. Nucleic Acids Res. 2015;43(Database issue):D743-D750. doi:10.1093/nar/gku1026 25355511 PMC4383884

[52] Bhoj EJ, Li M, Ahrens-Nicklas R, . Pathologic variants of the mitochondrial phosphate carrier SLC25A3: two new patients and expansion of the cardiomyopathy/skeletal myopathy phenotype with and without lactic acidosis. JIMD Rep. 2015;19(2):59-66. doi:10.1007/8904 25681081 PMC4501241

[53] Lim TKY, Rone MB, Lee S, Antel JP, Zhang J. Mitochondrial and bioenergetic dysfunction in trauma-induced painful peripheral neuropathy. Mol Pain. 2015;11(1):58. doi:10.1186/s12990-015-0057-7 26376783 PMC4574230

[54] Duggett NA, Griffiths LA, Flatters SJL. Paclitaxel-induced painful neuropathy is associated with changes in mitochondrial bioenergetics, glycolysis, and an energy deficit in dorsal root ganglia neurons. Pain. 2017;158(8):1499-1508. doi:10.1097/j.pain.0000000000000939 28541258 PMC5515641

[55] Yamashita A, Matsuoka Y, Matsuda M, Kawai K, Sawa T, Amaya F. Dysregulation of p53 and parkin induce mitochondrial dysfunction and leads to the diabetic neuropathic pain. Neuroscience. 2019;416:9-19. doi:10.1016/j.neuroscience.2019.07.045 31377450

[56] Gu Y, Qiu Z-L, Liu D-Z, . Differential gene expression profiling of the sciatic nerve in type 1 and type 2 diabetic mice. Biomed Rep. 2018;9(4):291-304. doi:10.3892/br.2018.1135 30233781 PMC6142038

[57] Perkins JR, Lees J, Antunes-Martins A, . PainNetworks: a web-based resource for the visualisation of pain-related genes in the context of their network associations. Pain. 2013;154(12):e2136560. doi:10.1016/j.pain.2013.09.003 24036287 PMC3863956

[58] Barry AM, Sondermann JR, Sondermann JH, Gomez-Varela D, Schmidt M. Region-resolved quantitative proteome profiling reveals molecular dynamics associated with chronic pain in the PNS and spinal cord. Front Mol Neurosci. 2018;11(August):259. doi:10.3389/fnmol.2018.00259 30154697 PMC6103001

[59] Dominguez E, Rivat C, Pommier B, Mauborgne A, Pohl M. JAK/STAT3 pathway is activated in spinal cord microglia after peripheral nerve injury and contributes to neuropathic pain development in rat. J Neurochem. 2008;107(1):50-60. doi:10.1111/j.1471-4159.2008.05566.x 18636982

[60] Li W, Wong CC, Zhang X, . *CAB39L* elicited an anti-Warburg effect via a LKB1-AMPK-PGC1α axis to inhibit gastric tumorigenesis. Oncogene. 2018;37(50):6383-6398. doi:10.1038/s41388-018-0402-1 30054562 PMC6296350

[61] Kwon M, Han J, Kim UJ, . Inhibition of mammalian target of rapamycin (mTOR) signaling in the insular cortex alleviates neuropathic pain after peripheral nerve injury. Front Mol Neurosci. 2017;10(March):79. doi:10.3389/fnmol.2017.00079 28377693 PMC5359287

