# Supplementary material for: Association of Genetic Variant at Chromosome 12q23.1 With Neuropathic Pain Susceptibility
Source: JAMA Netw Open. 2021 Dec 2;4(12):e2136560. doi: 10.1001/jamanetworkopen.2021.36560 (PMC8640893; doi:10.1001/jamanetworkopen.2021.36560)
Supplement: Supplement. — eMethods 1. Description of Study Cohorts eMethods 2. Self-completed Questionnaire Data eMethods 3. NP Phenotyping eMethods 4. Genotyping, Quality Control, and Imputation eMethods 5. Genome-Wide Association Analyses and Meta-analyses eMethods 6. In Silico Functional Annotation, Expression Quantitative Loci, and Colocalization Analysis eMethods 7. SNV-Based Heritability eReferences. eTable 1. Sensitivity and Specificity of Neuropathic Pain Phenotyping Methods in GoDARTS eTable 2. Most Significant SNVs (P < 5 × 10^-5^) Associated With NP in the Meta-analysis of All 3 Cohorts (GoDARTS, GS:SFHS, and UKBB) eTable 3. Sensitivity Analysis of the Most Significant SNVs Associated With NP in the Stage 2 Meta-analysis eTable 4. The Most Significant SNVs Associated With NP From the Overall Meta-analysis and Related Traits eTable 5. Expression Quantitative Trait Loci Information for the Most Signficant Genetic Loci Associated With NP in Human Dorsal Root Ganglia (eQTL DRG) and Brain Cortex (brain xQTL serve) eTable 6. Most Significant SNVs (P < 5 × 10^-5^) Associated With NP in Stage 1 (GoDARTS and GS:SFHS) and UKBB Study eTable 7. Association Results of Previously Reported SNVs in Each Study and Meta-analysis (Stage 1 and Stage 2) eTable 8. Combined Analysis of the Present Study Summary Statistics (Stage 1 and Stage 2) and the Original Study Summary Statistics eFigure 1. Quantile-Quantile Plot for the Results of Stage 2 Meta-analysis (GoDARTS, GS:SFHS, and UKBB) GWAS eFigure 2. Scattershot of Directly Typed Variant at Chromosome 12q23.1 From the UKBB Data eFigure 3. Multitissue eQTL Comparison for a Promising Candidate SNV for NP and Correlation With Expression of CAB39L eFigure 4. Plots Showing the P Value of Association Tests for SNVs With Possible NP in Stage 1 Meta-analysis (GoDARTS and GS:SFHS) eFigure 5. Regional Association Plot of an Index SNV in Stage 1 Meta-analysis (GoDARTS and GS:SFHS) [file jamanetwopen-e2136560-s001.pdf]

Supplemental Online Content

Veluchamy A, Hébert HL, van Zuydam NR, et al. Association of genetic variant at chromosome 12q23.1 with neuropathic pain susceptibility. *JAMA Netw Open.* 2021;4(12):e2136560. doi:10.1001/jamanetworkopen.2021.36560

eMethods 1. Description of Study Cohorts

eMethods 2. Self-completed Questionnaire Data

eMethods 3. NP Phenotyping

eMethods 4. Genotyping, Quality Control, and Imputation

eMethods 5. Genome-Wide Association Analyses and Meta-analyses

eMethods 6. In Silico Functional Annotation, Expression Quantitative Loci, and Colocalization Analysis

eMethods 7. SNV-Based Heritability

eReferences.

eTable 1. Sensitivity and Specificity of Neuropathic Pain Phenotyping Methods in GoDARTS

eTable 2. Most Significant SNVs ($P < 5 \times 10^{-5}$) Associated With NP in the Meta-analysis of All 3 Cohorts (GoDARTS, GS:SFHS, and UKBB)

eTable 3. Sensitivity Analysis of the Most Significant SNVs Associated With NP in the Stage 2 Meta-analysis

eTable 4. The Most Significant SNVs Associated With NP From the Overall Meta-analysis and Related Traits

eTable 5. Expression Quantitative Trait Loci Information for the Most Signficant Genetic Loci Associated With NP in Human Dorsal Root Ganglia (eQTL DRG) and Brain Cortex (brain xQTL serve)

eTable 6. Most Significant SNVs ($P < 5 \times 10^{-5}$) Associated With NP in Stage 1 (GoDARTS and GS:SFHS) and UKBB Study

eTable 7. Association Results of Previously Reported SNVs in Each Study and Meta-analysis (Stage 1 and Stage 2)

eTable 8. Combined Analysis of the Present Study Summary Statistics (Stage 1 and Stage 2) and the Original Study Summary Statistics

eFigure 1. Quantile-Quantile Plot for the Results of Stage 2 Meta-analysis (GoDARTS, GS:SFHS, and UKBB) GWAS

© 2021 Veluchamy A et al. *JAMA Network Open.*

eFigure 2. Scattershot of Directly Typed Variant at Chromosome 12q23.1 From the UKBB Data

eFigure 3. Multitissue eQTL Comparison for a Promising Candidate SNV for NP and Correlation With Expression of *CAB39L*

eFigure 4. Plots Showing the P Value of Association Tests for SNVs With Possible NP in Stage 1 Meta-analysis (GoDARTS and GS:SFHS)

eFigure 5. Regional Association Plot of an Index SNV in Stage 1 Meta-analysis (GoDARTS and GS:SFHS)

This supplemental material has been provided by the authors to give readers additional information about their work.

eMethods 1. Description of Study Cohorts

Genetics of Diabetes Audit and Research in Tayside Scotland (GoDARTS)

GoDARTS is a population-based cohort which aims to identify genetic factors influencing the risk of developing type 2 diabetes (T2D), response to treatment and diabetes related complications[1]. This study comprises 18,306 participants of European-heritage from Tayside, Scotland, of whom 10,149 participants have T2D, and 8,157 participants were diabetes-free at the time of recruitment. Genome-wide chip data are available for 7,857 T2D and 1,108 non-diabetes participants after quality control (QC) at the time of the present study. Median age of the cohort at baseline was 64 years. Participants provided consent for anonymous linkage of baseline and genetic data to routine electronic health records (EHR) including prescribing, laboratory data, mortality, hospital admissions and demography. This allows researchers to use this cohort as a longitudinal cohort for follow-up studies. Also, the participants provided consent to be re-contacted to participate in relevant studies in the future. This study was approved by the Tayside Committee for Medical Research (053/04).

Generation Scotland: Scottish Family Health Study (GS:SFHS)

GS:SFHS is a family-based study with socio-demographic, clinical and biological samples from 24,000 participants of European descent aged 18-98 years[2]. Participants were recruited between 2006 and 2011 through general medical practices across Scotland and if they had at least one first-degree relative aged 18 or more willing to participate. This study received ethical approval from the NHS Tayside committee on research ethics (05/S1401/89). This cohort was set up in order to identify and understand the contribution of genetic factors to major common complex diseases such as cardiovascular disease, diabetes, stroke, mental illness, and cognitive dysfunction. The median age of this cohort was 47.9 years at the time of recruitment. Whole-genome genotyping data are available for 20,032 participants after the QC. All participants provided broad consent to use their genetic data for a wide range of medical research, and for linkage of routine health care records and re-contact for future research purposes.

© 2021 Veluchamy A et al. *JAMA Network Open.*

United Kingdom Biobank (UKBB)

The UKBB is a population-based biomedical resource that aims to investigate the contribution of genetic and non-genetic determinants of diseases and outcomes to improve prevention, diagnosis, and treatment. This prospective cohort comprises 460,000 individuals of European ancestry from across the UK who were recruited 2006-2010 at age between 40 and 69 years. At the time of the recruitment, participants provided electronically signed consent to use their self-completed answers on socio-demographic, lifestyle, health-related information, and a range of physical measures. Also, participants reported if they were currently taking certain important classes of medication information through a touch-screen questionnaire followed by interview with a trained member of staff at the time of assessment centre. They provided the consent for their blood, urine, and saliva samples and longitudinal data through linkage of medical records including hospital inpatient data, death, and cancer register[3]. This cohort received ethical approval from the North West Multi-centre REC (11/NW/0382).

© 2021 Veluchamy A et al. *JAMA Network Open.*

eMethods 2. Self-completed Questionnaire Data

DOLORisk is an international collaboration involving members of established academic institutions and companies in Europe[4]. They designed a questionnaire based on an agreed approach to NP phenotyping by International consensus[5] (NeuroPPIC) led by the Neuropathic Pain Special Interest Group (NeuPSIG) of the International Association for the Study of Pain (IASP) to identify and characterise participants with NP. This self-completed questionnaire included information on pain history, pain medication, the severity of pain, quality of pain, pain location, pain interference, pain catastrophizing, health status, and quality of life, personality and lifestyle factors using validated questionnaire tools[4] DOLORisk Dundee[6] is a part of the DOLORisk consortium and is based on the two pre-existing population-based cohorts: Genetics of Diabetes Audit and Research in Tayside Scotland[1] andGeneration Scotland: Scottish Family Health Study[2]. Living participants of both the GoDARTS(N=5,236) and the GS:SFHS(N=20,221) who had given consent were contacted by mail with a letter of invitation, a Participant Information Leaflet (PIL) and DOLORisk paper questionnaire in optical character recognition (OCR) format labelled with a unique study code, along with a pre-paid return envelope through the Health Informatics Centre (HIC) (https://www.dundee.ac.uk/hic), a research support unit of the University of Dundee. The self-completed questionnaires from the participants were collected and managed by HIC through their secure mailing system and database. Questionnaire data were scanned, processed, and linked with anonymised participant IDs by HIC services for the DOLORisk study[6]. The confidential personal data in the questionnaire were stored securely and processed and entered into the data entry system. Data handling and delivery were conducted by HIC in a secure safe-haven environment to confirm data security and protection. Data were provided in flat file format and released on secure HIC servers for research purposes. Phenotype information was extracted from the questionnaire data and linked to pre-existing genetic and demographic data.

© 2021 Veluchamy A et al. *JAMA Network Open.*

eMethods 3. NP Phenotyping in the UKBB

GoDARTS and GS:SFHS:

Participants with chronic pain were identified using the following questions in the DOLORisk questionnaire: 1) “Are you currently troubled by pain or discomfort, either all the time or on and off?”; 2) “Are you currently taking medications specifically to treat pain or discomfort?”; and 3) “How long have you been suffering with this pain or discomfort?”. Participants were also asked to specify characteristics of the pain that bothered them the most, using a validated screening tool, the self-complete version of DN4 questionnaire which comprises seven items: burning, painful cold, electric shocks, tingling, pins and needles, numbness and itching. A positive response (“Yes”) to each item scored as 1, and negative response (“No”) scored as 0. Participants gave positive answers to either the first or second question or both and who reported a pain duration at least three months and scored at least 3 out of 7 on the DN4 questionnaire were classified as possible NP cases. Participants who gave a negative response to the question about current pain at the time of completing the questionnaire, and who were currently not taking pain medications were selected as controls for a case-control GWAS on NP.

UKBB

At the time of this study, direct neuropathic pain phenotyping information is not available in the UKBB. We therefore used the self-reported medical history information records as a proxy for NP phenotype. Dispensed medications information was captured from the answers given by the participants at an assessment centre through an interview with a trained nurse. Hospital admissions data, including the diagnosis associated with the reason for any admission, were extracted by linking to the available nation-wide participants’ electronic records. On the basis of NeuPSIG guidelines for NP treatment[7], the most relevant medications to include for case identification were gabapentin, pregabalin and duloxetine to identify individuals with likely NP. Duloxetine is used to treat depression, but it is not the first choice of drug for depression disorders treatment (National Institute for Health and Care Excellence. First-choice antidepressant use in adults with depression or generalised anxiety disorder. 2013;1–4). We did not have records of other commonly used medicines, capsaicin, and lidocaine plasters, for

© 2021 Veluchamy A et al. *JAMA Network Open.*

peripheral NP in the UKBB. Individuals who had no recorded history of having been prescribed any of these drugs were selected as controls for the GWAS. Apart from these drugs, subjects with a recorded history of amitriptyline, other tricyclic anti-depressants or tramadol were excluded from controls or cases, as these drugs are used to treat non-neuropathic pain and is not specific to NP. As gabapentin and pregabalin are used for epilepsy treatment[8], subjects were excluded from cases or controls if they had been admitted to hospital and formally diagnosed with epilepsy or if they had been recorded as receiving any of the following anti-epileptic medications: clobazam, clonazepam, eslicarbazepine, ethosuximide, lamotrigine, levetiracetam, lacosamide, perampanel, phenytoin, phenobarbital, sodium valproate, topiramate, and zonisamide. The International Classification of Diseases 10 (ICD-10) diagnosis codes, G40.0, G40.1, G40.2, G40.3, G40.4, G40.5, G40.6, G40.7, G40.8, G40.9, G41, and R56, were used to classify epilepsy and recurrent seizures in the hospital admissions records. These diagnosis codes were used to identify subjects with epilepsy in addition to their prescription history of gabapentin and pregabalin. Therefore, we have applied exclusion criteria to avoid possible misclassification bias. Moreover, cases and controls were matched for ancestry, and principal components to address any differences.

eMethods 4. Genotyping, Quality Control, and Imputation

Genotype data for the GoDARTS[1], GS:SFHS[9] and UKBB[10] study populations were pre-existing and linked to the phenotype data. Blood samples were collected from the GoDARTS participants and used for genotyping by either Affymetrix 6.0 or Illumina Omni Express chips or Illumina Infinimum Broad chips. Samples were excluded based on the following criteria: samples with a call rate less than 95%, the mismatch between clinical data and genotypic gender, batch effects, ancestry outliers using principal components, sample duplicates (IBD score > 0.8). The poor-quality markers were identified and excluded on the basis of monomorphism, Hardy-Weinberg Equilibrium (HWE) p-value less than 1×10^{-6} and call rate less than 95%. PLINK 1.07[11] was used to perform the quality assessment for genotyping data from all platforms. Blood or saliva were collected from the GS:SFHS participants to extract DNA. The samples were genotyped on the Illumina Human Omni Express Exome-8 v1.0 Bead chip, and Illumina Omni Express Exome-8 v1.2 Bead Chip. Quality control assessment was performed for genotyping data using GenABEL 1.7-6[12] and PLINK 1.07[11]. Samples were removed if they met the following criteria: samples with a call rate less than 98%, sample duplicates, and samples with gender discrepancies between reported and genotype data. SNVs with a call rate less than 98% HWE p-value less than 1×10^{-6}. and MAF < 1%. Ancestry outliers were identified by applying a six standard deviation cut-off in a principal component analysis using genotyping data from the GS:SFHS participants merged with 1,092 individuals from the 1,000 Genomes project[13]. For the UKBB cohort, blood samples were collected to extract DNA from the participants on their visit to the UKBB assessment centre. Genome-wide genotyping was performed using two similar custom-designed genotyping arrays including UK Biobank Axiom (438,427 participants) and UK BiLEVE Axiom Affymetrix array (49,950 participants)[10]. UKBB's genotyping, QC, PCA and imputation methodology are described in detail elsewhere[3]. We selected individuals of European ancestry in the UKBB based on principal component analysis (PCA) and self-reporting ancestry information.

The genotype data from all three cohorts were imputed against a haplotype reference consortium (HRC r1.1) reference panel in NCBI build 37[14]. Post-imputation QC checks were conducted in all individual studies; monomorphic markers or those with imputation quality score < 0.4 were excluded. The genomic position of the markers is based on the NCBI human genome build 37.

© 2021 Veluchamy A et al. *JAMA Network Open.*

eMethods 5. Genome-Wide Association Analyses and Meta-analyses

We conducted genome-wide association analyses in each of the three cohorts (GoDARTS, GS:SFHS and UKBB) separately. Both genotypic and imputed markers were tested for their association with NP using a linear mixed non-infinitesimal model in BOLT-LMM software to account for relatedness and population structure[15]. This model assumes an additive genetic model that was corrected for age and gender. The beta estimates and SEs were converted and approximated to traditional odds ratios using the formula below (https://data.broadinstitute.org/alkesgroup/BOLT-LMM/).

$$\log OR = {}^{\beta}\!/_{(\mu * (1-\mu))}$$

μ denotes case proportion.

$$SE_{beta} = {}^{SE}\!/_{(\mu * (1-\mu))}$$

We conducted the meta-analysis of GWAS (GoDARTS and GS:SFHS) in stage1 using a fixed effect inverse variance weighted meta-analysis in GWAMA[16]. The genomic control inflation factor lambda was 1.023. To increase study power, we combined the summary results from all three cohorts in stage2. We calculated genomic inflation factors (ʎ) in individual data sets for population stratification and applied genomic control. Prior to the meta-analysis, SNVs with low minor allele frequency (< 0.001), low imputation quality score (<0.4) and deviation from Hardy-Weinberg equilibrium ($P<1\times10^{-6}$) were removed from the summary GWAS results. The presence of heterogeneity between these studies was examined with the I^2 statistic. Manhattan, Quantile-quantile (QQ) and forest plots were generated to visualize the GWAS results using R 3.4 and metafor R package[17]. Regional association plots were created using LocusZoom[18]. ScatterShot is a web application which was used to generate cluster plot images for directly typed variants in the from the UKBB dataset[19].

© 2021 Veluchamy A et al. *JAMA Network Open.*

eMethods 6. In-silico Functional Annotation, Expression Quantitative Loci, and Colocalization Analysis

Variants were annotated using the University of California Santa Cruz (UCSC) Genome resource[20] based on the Genome Reference Consortium Human genome build 37. Functional annotations of the significant SNVs and genetic risk loci were identified using functional mapping and annotation of genome-wide association studies[21] (FUMA) which includes the annotation databases such as RegulomeDB[22], HaploReg v1[23] and Chromatin states[24]. ChromHMM state for 127 tissues/cell types indicates the functional effects of gene expression using expression quantitative trait loci (eQTLs) of various tissue types and chromatin interactions using Hi-C. The FINEMAP package was used to identify specific genetic variants that are likely to be causal from the summary statistics of the SNVs at the most significant locus by applying shotgun stochastic search algorithm[25].

The Genotype-Tissue Expression (GTEx) v7[26] database allow users to view gene expression data and eQTL results, and provides a controlled access system for de-identified individual-level genotype and clinical data. The GTEx project provides a resource that contains information about the relationship between human gene expression and genetic variation by analysing genotype and expression data obtained from multiple human tissues from donors. A recent study by Parisien et al. reported a database of eQTLs in a collection of human dorsal root ganglia and the association of dorsal root ganglion (DRG) eQTLs with pain-related genetic association results[27]. They also reported eGenes in DRGs, overlapping of DRG eQTLs with cis-eQTLs in brain and blood, and the association of HLA gene loci for DRG eQTLs and pain phenotypes. This can be used for interpreting human GWAS results with sensory components. The eQTL data of DRGs (https://humanpaingenetics.org/DRG-eQTLs/) are freely available online for downstream analysis GWAS focussed on pain and other sensory phenotypes. Brain xQTL serve database provides information about the association between genetic variants and molecular traits derived from the brain cortex[28] (mostafavilab.stat.ubc.ca/xqtl).

© 2021 Veluchamy A et al. *JAMA Network Open.*

Co-localisation analyses were conducted to test co-localisation between the expression of eQTL for brain tissues from GTEx v6 and the most significant SNV from this study using the R package "*coloc*" which is based on Bayesian statistical methods and generates five posterior probabilities (PP0, PP1,PP2,PP3,PP4) for each locus[29]. We report the gene with the highest probability score (PP4) of being correlated with the most significant signal.

In Silico lookups for the most significant SNVs using GeneATLAS[30] database to examine the association of pain related traits in the UKBB. It is a large database containing genetic association results for 118 quantitative and 660 case-control traits of 452,264 UKBB participants of European heritage.

© 2021 Veluchamy A et al. *JAMA Network Open.*

eMethods 7. SNV-Based Heritability

SNV-based heritability of NP was estimated from GWAS summary statistics using Linkage Disequilibrium Score Regression (LDSC) (https://github.com/bulik/ldsc) software which accounts for linked markers and expects that each marker contributes equally to the phenotypic variance. The LDSC utilises SNVs across the whole genome that passed the imputation quality score, and strand ambiguity and slope from $\chi 2$ statistics regressed on GWAS SNVs' LD scores. It does not require an individual study genotype data. We used full summary statistics data from the meta-analysis of GWAS to estimate the SNV-based heritability in a liability scale [31].

© 2021 Veluchamy A et al. *JAMA Network Open.*

eReferences.

1. Hébert HL, Shepherd B, Milburn K, et al. Cohort Profile: Genetics of Diabetes Audit and Research in Tayside Scotland (GoDARTS). *Int J Epidemiol*. 2017:1-12. doi:10.1093/ije/dyx140
2. Smith BH, Campbell A, Linksted P, et al. Cohort profile: Generation scotland: Scottish family health study (GS: SFHS). The study, its participants and their potential for genetic research on health and illness. *Int J Epidemiol*. 2013;42(3):689-700. doi:10.1093/ije/dys084
3. Bycroft C, Freeman C, Petkova D, et al. The UK Biobank resource with deep phenotyping and genomic data. *Nature*. 2018;562(7726):203-209. doi:10.1038/s41586-018-0579-z
4. Pascal MMV, Themistocleous AC, Baron R, et al. DOLORisk: Study protocol for a multi-centre observational study to understand the risk factors and determinants of neuropathic pain [version 2; referees: 2 approved]. *Wellcome Open Res*. 2019;3:1-23. doi:10.12688/wellcomeopenres.14576.2
5. Hecke O Van, Kamerman PR, Attal N, et al. Neuropathic pain phenotyping by international consensus (NeuroPPIC) for genetic studies : a NeuPSIG systematic review , Delphi survey , and expert panel recommendations. *Pain*. 2015;156(11):2337-2353. doi:10.1097/j.pain.0000000000000335
6. Hébert HL, Veluchamy A, Baskozos G, et al. Cohort profile : DOLORisk Dundee : a longitudinal study of chronic neuropathic pain. *BMJ Open*. 2021:1-11. doi:10.1136/bmjopen-2020-042887
7. Finnerup NB, Attal N, Haroutounian S, et al. Pharmacotherapy for neuropathic pain in adults: A systematic review and meta-analysis. *Lancet Neurol*. 2015;14(2):162-173. doi:10.1016/S1474-4422(14)70251-0
8. Hamandi K, Sander JW. Pregabalin: A new antiepileptic drug for refractory epilepsy. *Seizure*. 2006;15(2):73-78. doi:10.1016/j.seizure.2005.11.005
9. Smith BH, Campbell H, Blackwood D, et al. Generation Scotland: The Scottish Family Health Study; a new resource for researching genes and heritability. *BMC Med Genet*. 2006;7. doi:10.1186/1471-2350-7-74
10. Bycroft C, Freeman C, Petkova D, et al. Genome-wide genetic data on 500,000 UK Biobank participants. *bioRxiv*. 2017:166298. doi:10.1101/166298
11. Purcell S, Neale B, Todd-Brown K, et al. PLINK: A Tool Set for Whole-Genome Association and Population-Based Linkage Analyses. *Am J Hum Genet*. 2007;81(3):559-575. doi:10.1086/519795
12. Aulchenko YS, Ripke S, Isaacs A, van Duijn CM. GenABEL: An R library for genome-wide association analysis. *Bioinformatics*. 2007;23(10):1294-1296. doi:10.1093/bioinformatics/btm108
13. Nagy R, Boutin TS, Marten J, et al. Exploration of haplotype research consortium

© 2021 Veluchamy A et al. *JAMA Network Open.*

imputation for genome-wide association studies in 20,032 Generation Scotland participants. *Genome Med*. 2017;9(1):1. doi:10.1186/s13073-017-0414-4

14. McCarthy S, Das S, Kretzschmar W, et al. A reference panel of 64,976 haplotypes for genotype imputation. *Nat Genet*. 2016;48(10):1279-1283. doi:10.1038/ng.3643

15. Loh PR, Tucker G, Bulik-Sullivan BK, et al. Efficient Bayesian mixed-model analysis increases association power in large cohorts. *Nat Genet*. 2015;47(3):284-290. doi:10.1038/ng.3190

16. Magi R, Morris AP. GWAMA: software for genome-wide association meta-analysis. *BMC Bioinformatics*. 2010;11(ii):288. doi:10.1186/1471-2105-11-288

17. Veichtbauer W. Conducting meta-analyses in R with the metafor package. *J Stat Softw*. 2010;36(3):1-48.

18. Pruim RJ, Welch RP, Sanna S, et al. LocusZoom: Regional visualization of genome-wide association scan results. *Bioinformatics*. 2011;27(13):2336-2337. doi:10.1093/bioinformatics/btq419

19. McCarthy Group. ScatterShot.

20. Speir ML, Zweig AS, Rosenbloom KR, et al. The UCSC Genome Browser database: 2016 update. *Nucleic Acids Res*. 2015;44(D1):D717-D725. doi:10.1093/nar/gkv1275

21. Watanabe K, Taskesen E, Van Bochoven A, Posthuma D. Functional mapping and annotation of genetic associations with FUMA. *Nat Commun*. 2017;8(1):1-10. doi:10.1038/s41467-017-01261-5

22. Boyle AP, Hong EL, Hariharan M, et al. Annotation of functional variation in personal genomes using RegulomeDB. *Genome Res*. 2012;22(9):1790-1797. doi:10.1101/gr.137323.112

23. Ward LD, Kellis M. HaploReg: A resource for exploring chromatin states, conservation, and regulatory motif alterations within sets of genetically linked variants. *Nucleic Acids Res*. 2012;40(D1):1-5. doi:10.1093/nar/gkr917

24. Ernst J, Kellis M. ChromHMM: automating chromatin state discovery and characterization. *Nat Methods*. 2009;9(3):215-216. doi:10.1016/j.neuron.2009.10.017.A

25. Benner C, Spencer CCA, Havulinna AS, Salomaa V, Ripatti S, Pirinen M. FINEMAP: Efficient variable selection using summary data from genome-wide association studies. *Bioinformatics*. 2016;32(10):1493-1501. doi:10.1093/bioinformatics/btw018

26. Ardlie KG, Deluca DS, Segre A V., et al. The Genotype-Tissue Expression (GTEx) pilot analysis: Multitissue gene regulation in humans. *Science (80-)*. 2015;348(6235):648-660. doi:10.1126/science.1262110

27. Parisien M, Khoury S, Chabot-Doré AJ, et al. Effect of Human Genetic Variability on Gene Expression in Dorsal Root Ganglia and Association with Pain

Phenotypes. *Cell Rep*. 2017;19(9):1940-1952. doi:10.1016/j.celrep.2017.05.018

28. Ng B, White CC, Klein HU, et al. An xQTL map integrates the genetic architecture of the human brain's transcriptome and epigenome. *Nat Neurosci*. 2017;20(10):1418-1426. doi:10.1038/nn.4632

29. Giambartolomei C, Vukcevic D, Schadt EE, et al. Bayesian Test for Colocalisation between Pairs of Genetic Association Studies Using Summary Statistics. *PLoS Genet*. 2014;10(5). doi:10.1371/journal.pgen.1004383

30. Canela-Xandri O, Rawlik K, Tenesa A. An atlas of genetic associations in UK Biobank. *Nat Genet*. 2018;50(November):1593-1599. doi:10.1101/176834

31. Speed D, Balding DJ, Dk. Better estimation of SNP heritability from summary statistics provides a new understanding of the genetic architecture of complex traits. *bioRxiv*. 2018. doi:10.1101/284976

© 2021 Veluchamy A et al. *JAMA Network Open.*

eTable 1. Sensitivity and Specificity of Neuropathic Pain Phenotyping Methods in GoDARTS

	DOLORisk questionnaire-based phenotyping			
Prescribing-based phenotyping	**Neuropathic pain phenotype**	**Cases**	**Controls**	**Total**
	**Cases**	257	44	301
	**Controls**	63	358	421
	**Total**	320	402	
	**Sensitivity = 80.3%**<br>**Specificity = 89.0%**			

© 2021 Veluchamy A et al. *JAMA Network Open.*

eTable 2. Most Significant SNVs (*P* < 5 × 10^{-5}) Associated With NP in the Meta-analysis of All 3 Cohorts (GoDARTS, GS:SFHS and UKBB)

RSID	CHR	POS	EA	NEA	EAF	OR	OR_95L	OR_95U	P	I^2	GENE
rs369920026	12	98585582	A	G	0.005	1.683	1.404	2.023	1.30×10^{-8}	0.00	*SLC25A3*
rs185663675	12	98602402	A	G	0.006	1.612	1.350	1.924	5.46×10^{-8}	0.02	*SLC25A3*
rs17027891	12	98581935	C	T	0.007	1.602	1.351	1.901	7.50×10^{-8}	0.10	*SLC25A3*
rs17027910	12	98586595	A	G	0.007	1.564	1.324	1.852	1.90×10^{-7}	0.00	*SLC25A3*
rs7992766	13	49905672	A	C	0.747	1.091	1.054	1.141	1.22×10^{-7}	0.31	*CAB39L*
rs150900085	3	135174848	C	T	0.007	1.580	1.321	1.883	3.04×10^{-7}	0.04	*EPHB1*
rs148034142	2	52976279	C	T	0.993	0.546	0.429	0.695	1.50×10^{-6}	-	*CHAC2*
rs77526294	8	49946568	G	A	0.944	1.176	1.103	1.255	1.54×10^{-6}	0.00	*SNAI2*
rs4331318	15	91582257	C	T	0.948	0.853	0.799	0.909	2.01×10^{-6}	0.05	*VPS33B*
rs7336018	13	49906514	C	T	0.759	1.090	1.053	1.129	2.11×10^{-6}	0.48	*CAB39L*
rs78726778	6	65985758	T	C	0.933	0.863	0.813	0.916	2.24×10^{-6}	0.71	*EYS*
rs79154996	6	25736698	G	A	0.975	0.781	0.707	0.864	2.26×10^{-6}	0.35	*HIST1H2AA*
rs79154996	6	25736698	G	A	0.975	0.781	0.707	0.864	2.26×10^{-6}	0.35	*HIST1H2BPS1*
rs79154996	6	25736698	G	A	0.975	0.781	0.707	0.864	2.26×10^{-6}	0.35	*HIST1H2BA*
rs7335286	13	49897739	C	A	0.248	0.918	0.887	0.951	2.53×10^{-6}	0.51	*CAB39L*
rs7322021	13	49903046	A	G	0.747	1.088	1.051	1.126	2.64×10^{-6}	0.51	*CAB39L*
rs145804345	12	98690315	C	T	0.994	0.631	0.523	0.761	2.66×10^{-6}	0.00	*SLC9A7P1*
rs7985932	13	49911527	A	T	0.759	1.089	1.052	1.128	2.70×10^{-6}	0.47	*CAB39L*
rs61692854	15	91582496	C	T	0.947	0.856	0.803	0.912	3.11×10^{-6}	0.00	*VPS33B*
rs13210851	6	65906043	C	T	0.951	0.847	0.792	0.907	3.18×10^{-6}	0.41	*EYS*
rs9535201	13	49897577	A	G	0.763	1.089	1.051	1.127	3.59×10^{-6}	0.47	*CAB39L*
rs115353340	1	2882483	C	T	0.988	0.704	0.608	0.815	4.47×10^{-6}	0.52	*56kb 5' of ACTRT2*
rs34932751	6	25762241	T	C	0.974	0.793	0.720	0.874	4.55×10^{-6}	0.29	*SLC17A4*
rs4688956	4	4343240	C	T	0.421	0.931	0.904	0.959	4.87×10^{-6}	0.00	-
rs72980761	6	138175666	G	A	0.847	0.906	0.869	0.944	5.04×10^{-6}	0.00	-
rs9535202	13	49901738	A	C	0.759	1.086	1.049	1.125	5.17×10^{-6}	0.50	*CAB39L*
rs138847726	4	13020910	C	T	0.964	1.207	1.115	1.307	5.24×10^{-6}	0.00	-
rs7992582	13	49905581	A	G	0.758	1.086	1.049	1.125	5.28×10^{-6}	0.45	*CAB39L*
rs75616385	12	70527056	G	C	0.910	0.887	0.843	0.933	5.28×10^{-6}	0.00	*CNOT2/KCNMB4*
rs4689309	4	4343725	A	T	0.421	0.931	0.904	0.960	5.34×10^{-6}	0.00	-
rs150309683	16	70284053	T	C	0.972	0.800	0.729	0.879	5.36×10^{-6}	0.10	*EXOSC6*
rs150309683	16	70284053	T	C	0.972	0.800	0.729	0.879	5.36×10^{-6}	0.10	*AARS*

© 2021 Veluchamy A et al. *JAMA Network Open.*

RSID	CHR	POS	EA	NEA	EAF	OR	OR_95L	OR_95U	P	I^2	GENE
rs12578473	12	70530616	G	T	0.910	0.887	0.843	0.933	5.92×10^{-6}	0.00	*CNOT2/KCNMB4*
rs78979790	12	70533571	A	G	0.910	0.887	0.844	0.933	6.14×10^{-6}	0.00	*CNOT2/KCNMB4*
rs112906219	6	138212862	C	T	0.843	0.908	0.871	0.946	6.36×10^{-6}	0.00	*TNFAIP3*
rs77684790	12	70499715	C	T	0.910	0.888	0.844	0.934	6.36×10^{-6}	0.00	*CNOT2/KCNMB4*
rs76712326	12	70501362	T	C	0.910	0.888	0.844	0.934	6.42×10^{-6}	0.00	*CNOT2/KCNMB4*
rs960591	12	70512598	A	G	0.910	0.888	0.844	0.934	6.59×10^{-6}	0.00	*CNOT2/KCNMB4*
rs4761295	12	70505474	T	C	0.910	0.888	0.844	0.934	6.60×10^{-6}	0.00	*CNOT2/KCNMB4*
rs74438250	12	70507017	T	G	0.910	0.888	0.844	0.934	6.65×10^{-6}	0.00	*CNOT2/KCNMB4*
rs77275828	12	70501844	C	T	0.910	0.888	0.844	0.934	6.67×10^{-6}	0.00	*CNOT2/KCNMB4*
rs79376147	12	70512178	T	A	0.910	0.888	0.844	0.934	6.68×10^{-6}	0.00	*CNOT2/KCNMB4*
rs4547147	12	70506188	G	A	0.910	0.888	0.844	0.934	6.75×10^{-6}	0.00	*CNOT2/KCNMB4*
rs78244236	12	70503212	A	G	0.911	0.888	0.844	0.934	6.82×10^{-6}	0.00	*CNOT2/KCNMB4*
rs7335804	13	49897815	G	A	0.237	0.921	0.889	0.954	7.03×10^{-6}	0.45	*CAB39L*
rs79512135	12	70501072	T	C	0.910	0.888	0.845	0.934	7.25×10^{-6}	0.00	*CNOT2/KCNMB4*
rs78313276	12	70510115	C	T	0.910	0.888	0.845	0.935	7.38×10^{-6}	0.00	*CNOT2*
rs12775058	10	12240680	C	T	0.973	0.794	0.720	0.876	7.42×10^{-6}	0.00	*NUDT5*
rs12775058	10	12240680	C	T	0.973	0.794	0.720	0.876	7.42×10^{-6}	0.00	*CDC123*
rs56364844	15	91586112	T	C	0.948	0.859	0.805	0.917	7.43×10^{-6}	0.00	-
rs141456350	12	70510197	C	A	0.910	0.889	0.845	0.935	7.45×10^{-6}	0.00	*CNOT2*
rs2118427	12	70508552	C	T	0.910	0.889	0.845	0.935	7.56×10^{-6}	0.00	*CNOT2*
rs75267777	12	70501154	A	G	0.910	0.889	0.845	0.935	7.61×10^{-6}	0.00	*CNOT2*
rs71507307	9	12233221	G	A	0.964	1.206	1.113	1.307	7.96×10^{-6}	0.00	-
rs72761306	15	91584219	G	A	0.948	0.860	0.807	0.918	8.06×10^{-6}	0.00	-
rs1025692	12	70513806	A	G	0.910	0.889	0.845	0.935	8.19×10^{-6}	0.00	*CNOT2//KCNMB4*
rs112990863	3	88714964	T	A	0.007	1.461	1.243	1.721	8.99×10^{-6}	0.85	*EPHA3*

CHR, chromosome; EA, effect allele; EAF, Effect allele frequency; I^2, heterogeneity measure; N, number of samples; NEA, noneffect allele; OR, odds ratio; OR_95L, 95% lower confidence interval; OR_95U, 95% upper confidence interval; POS, base position based on NCBI build 37; GoDARTS, Genetics of Diabetes Audit and Research in Tayside Scotland; GS:SFHS, Generation Scotland: Scottish Family Health Study; Stage 1 meta-analysis; UKBB, United Kingdom BioBank.

© 2021 Veluchamy A et al. *JAMA Network Open.*

eTable 3. Sensitivity Analysis of the Most Significant SNVs Associated With NP in the Stage 2 Meta-analysis

**SNVs**	GS:SFHS + UKBB		GoDARTS + UKBB	
	OR (95% CI)	P	OR (95% CI)	P
rs369920026	1.27(1.15-1.41)	2.48×10^{-8}	1.28(1.11-1.48)	1.63×10^{-4}
rs185663675	1.25(1.13-1.39)	1.86×10^{-7}	1.28(1.11-1.47)	7.56×10^{-4}
rs17027891	1.24(1.12-1.37)	1.34×10^{-7}	1.31(1.13-1.51)	1.56×10^{-4}
rs17027910	1.23(1.11-1.37)	1.71×10^{-7}	1.27(1.08-1.49)	1.58×10^{-4}
rs7992766	1.01(1.06-1.14)	2.27×10^{-7}	1.08(1.02-1.14)	6.2×10^{-3}
rs112990863	1.19(1.07-1.13)	4.88×10^{-6}	1.01(0.77-1.27)	0.897

GoDARTS, Genetics of Diabetes Audit and Research in Tayside Scotland; GS:SFHS, Generation Scotland: Scottish Family Health Study.

© 2021 Veluchamy A et al. *JAMA Network Open.*

eTable 4. The Most Significant SNVs Associated With NP From The Overall Meta-analysis and Related Traits

NP-associated SNV/Gene	Relevant traits association	Effect allele	OR / Beta	P-value	Source
rs369920026 / rs185663675 *SLC25A3*	Viral hepatitis	A	3.34 / 0.002	0.001	GeneATLAS database using UKBB[30]
	Disc problem	A	1.23 / 0.003	0.04	
	Fibromyalgia	A	0.48 / -0.001	0.04	
rs17027891/ rs17027910 *SLC25A3*	Disc problem	C	1.28/ 0.004	0.01	GeneATLAS database using UK Biobank[30]
	Fibromyalgia	C	0.47/-0.001	0.02	
rs7992766 / *CAB39L*	Lymphocyte count	C	NA / -0.004	0.0002	GeneATLAS database using UK Biobank[30]
	Alcohol intake frequency	C	NA / -0.011	0.0004	
	Ulcer of lower limb	C	0.858 / -0.0003	0.0014	
	Disorders of brain	C	0.907/ -0.0003	0.01	
	Neck/shoulder pain for 3+ months	C	0.961 / -0.039	0.0012	UK Biobank Neale v2 (2018) (http://www.nealelab.is/uk-biobank)
rs150900085 / *EPHB1*	Back pain	C	1.49 / -0.001	0.066	GeneATLAS database using UK Biobank[30]
	Pain and other conditions associated with female genital organs and menstrual cycle	C	1.52 / 0.006	0.007	
	Disorders of lipoprotein metabolism	C	0.886/0.009	0.0089	
	Sciatica	C	0.767/0.002	0.064	
	Headaches for 3+ months	C	1.22/0.195	0.005	

© 2021 Veluchamy A et al. *JAMA Network Open.*

eTable 5. Expression Quantitative Trait Loci Information for the Most Signficant Genetic Loci Associated With NP in Human Dorsal Root Ganglia (eQTL DRG) and Brain Cortex (brain xQTL serve)

SNV	TagSNV	Beta	P-value	Gene	Tissue	Association with NP SNV / Tag SNV
rs7334929:T:G	rs7992766:A:C	-0.23	8.9×10^{-07}	*CDADC1/ CAB39L*	DRG	0.002 / 1.22×10^{-07}
rs7992766:A:C				*CAB39L*	Brain Cortex	8.51×10^{-21}
rs10049228:T:C	rs11712544:G:A	0.12	4.9×10^{-05}	*EPHB1*	DRG	0.009/0.006

DRG, dorsal root ganglia.

© 2021 Veluchamy A et al. *JAMA Network Open.*

eTable 6. Most Significant SNVs (*P* < 5 × 10^{-5}) Associated With NP in Stage 1 (GoDARTS and GS:SFHS) and UKBB Study

RSID	CHR	POS	EA / NEA	EAF	Study	OR	OR_95L	OR_95U	P value	I^2	N	GENE
					Stage 1	1.743	1.434	2.112	3.73×10^{-08}		3,978	
**rs112990863**	3	88714964	T/A	0.009	UKBB	1.008	0.757	1.342	0.960	0.20	428,925	*EPHA3*
					Stage 1	0.546	0.436	0.685	1.59×10^{-07}		3,978	
**rs150675307**	2	100319170	T/G	0.991	UKBB	1.309	0.955	1.794	0.094	0.00	428,925	*AFF3*
					Stage 1	0.736	0.656	0.826	3.09×10^{-07}		3,978	
**rs182827559**	3	140357428	T/C	0.968	UKBB	0.993	0.863	1.143	0.911	0.00	428,925	*TRIM42*
					Stage 1	0.549	0.438	0.689	3.61×10^{-07}	0.00	3,273	
**rs145943613**	3	88731531	T/G	0.992	UKBB	1.079	0.786	1.482	0.640		428,925	*EPHA3*
					Stage 1	0.752	0.672	0.841	9.91×10^{-07}		3,978	
**rs72977016**	3	140370012	C/T	0.967	UKBB	0.981	0.864	1.137	0.884	0.00	428,925	*TRIM42*
					Stage 1	0.706	0.615	0.809	1.05×10^{-06}	0.16	3,978	
**rs141384665**	12	91136799	T/G	0.979	UKBB	0.984	0.824	1.191	0.948		428,925	*66kb 5' of RP11-20L19.1*
					Stage 1	1.671	1.342	2.071	1.31×10^{-06}	0.00	4,076	
**rs150900085**	3	135174847	C/T	0.007	UKBB	1.401	1.023	1.923	**0.034**		428,925	*EPHB1*
					Stage 1	0.880	0.836	0.926	1.44×10^{-06}		4,076	
**rs4648390**	1	2700372	C/T	0.825	UKBB	0.971	0.911	1.038	0.410	0.00	428,925	*TTC34*
					Stage 1	0.444	0.321	0.614	1.46×10^{-06}		3,655	
**rs182181935**	9	13277439	G/T	0.996	UKBB	1.461	0.904	2.361	0.121	0.15	428,925	*MPDZ*
					Stage 1	0.801	0.732	0.875	1.62×10^{-06}		4,076	
**rs74546839**	3	140259531	T/C	0.952	UKBB	0.940	0.850	1.060	0.350	0.12	428,925	*CLSTN2*

© 2021 Veluchamy A et al. *JAMA Network Open.*

RSID	CHR	POS	EA / NEA	EAF	Study	OR	OR_95L	OR_95U	P value	I^2	N	GENE
**rs28647750**	1	2632016	C/G	0.826	Stage 1	0.880	0.836	0.926	1.68×10^{-06}		4,076	*TTC34*
					UKBB	0.962	0.901	1.033	0.320	0.00	428,925	
**rs7992766**	13	49905672	A/C	0.750	Stage 1	1.097	1.051	1.145	2.41×10^{-05}	0.23	3,978	*CAB39L*
					UKBB	1.097	1.038	1.159	9.00×10-04		428,925	

CHR, chromosome; EA, effect allele; EAF, Effect allele frequency; I^2, heterogeneity measure; N, number of samples; NEA, noneffect allele; OR, odds ratio; OR_95L, 95% lower confidence interval; OR_95U, 95% upper confidence interval; POS, base position based on NCBI build 37; GoDARTS, Genetics of Diabetes Audit and Research in Tayside Scotland; GS:SFHS, Generation Scotland: Scottish Family Health Study; Stage 1, meta-analysis of GoDARTS and GS:SFHS GWAS; UKBB, United Kingdom BioBank.

© 2021 Veluchamy A et al. *JAMA Network Open.*

eTable 7. Association Results of Previously Reported SNVs in Each Study and Meta-analysis (Stage 1 and Stage 2)

RSID	CHR	POS	EA	NEA	EAF	Study	OR	OR_95L	OR_95U	P_{unadj}	I^2	GENE
rs1901531	15	45005381	T	C	0.817	Stage 1	0.804	0.899	0.991	**0.023**	0.0	*B2M*
						UKBB	1.002	0.939	1.069	0.951		
						Stage 2	0.964	0.928	1.003	**0.041**	0.1	
rs6986153	8	108072044	G	A	0.207	Stage 1	0.981	0.936	1.028	0.427	0.2	*HMGB1P46*
						UKBB	0.938	0.884	0.995	**0.034**		
						Stage 2	0.964	0.929	1.000	0.057	0.2	
rs267206	6	7860815	C	T	0.185	Stage 1	0.975	0.928	1.025	0.330	0.0	*BMP6*
						UKBB	0.947	0.890	1.008	0.086		
						Stage 2	0.964	0.927	1.002	0.071	0.0	
rs1800629	6	31543031	G	A	0.803	Stage 1	NA	NA	NA	NA		*TNF-A*
						UKBB	1.056	0.994	1.122	0.076		
						Stage 2	1.056	0.994	1.122	0.076	0.0	
rs7033149	9	32398234	G	T	0.142	Stage 1	1.071	1.014	1.129	**0.015**	0.0	*ACO1*
						UKBB	0.982	0.917	1.053	0.622		
						Stage 2	1.036	0.993	1.081	0.106	0.1	
rs887797	17	64579445	G	A	0.679	Stage 1	1.005	0.965	1.047	0.796	0.6	*PRKCA*
						UKBB	1.053	0.999	1.108	**0.051**		
						Stage 2	1.023	0.991	1.056	0.165	0.6	
rs71647933	1	33945601	A	G	0.825	Stage 1	0.967	0.919	1.017	0.201	0.0	*ZSCAN20*
						UKBB	0.979	0.919	1.044	0.520		
						Stage 2	0.972	0.934	1.011	0.166	0.0	
rs4680	22	19951271	G	A	0.484	Stage 1	0.952	0.917	0.989	**0.013**	0.5	*COMT*
						UKBB	1.021	0.976	1.074	0.321		
						Stage 2	0.979	0.951	1.009	0.176	0.7	
rs12478318	2	167133540	T	G	0.996	Stage 1	0.806	0.591	1.103	0.186	0.0	*SCN9A*
						UKBB	1.027	0.617	1.712	0.920		
						Stage 2	0.807	0.590	1.102	0.187	0.0	
rs4369876	2	167129256	C	A	0.996	Stage 1	0.806	0.591	1.103	0.186	0.0	*SCN9A*
						UKBB	1.098	0.671	1.797	0.711		
						Stage 2	0.807	0.590	1.102	0.187	0.0	
rs1800795	7	22766645	C	G	0.430	Stage 1	0.995	0.956	1.034	0.787	0.0	*IL6*
						UKBB	1.062	1.012	1.115	**0.014**		
						Stage 2	1.021	0.990	1.052	0.196	0.4	
rs16966334	15	45003114	C	G	0.976	Stage 1	0.927	0.822	1.044	0.221	0.0	*B2M*
						UKBB	0.960	0.821	1.123	0.611		
						Stage 2	0.939	0.854	1.033	0.203	0.0	

© 2021 Veluchamy A et al. *JAMA Network Open.*

RSID	CHR	POS	EA	NEA	EAF	Study	OR	OR_95L	OR_95U	P_{unadj}	I^2	GENE
rs8007267	14	55378991	C	T	0.822	Stage 1	1.045	0.994	1.099	0.091	0.6	*GCH1*
						UKBB	1.001	0.937	1.062	0.932		
						Stage 2	1.026	0.987	1.067	0.208	0.6	
rs3024505	1	206939904	G	A	0.846	Stage 1	1.041	0.988	1.097	0.137	0.0	*IL10*
						UKBB	1.002	0.930	1.062	0.864		
						Stage 2	1.023	0.982	1.066	0.292	0.0	
rs13072552	3	148913126	G	T	0.922	Stage 1	0.960	0.893	1.032	0.283		*CP*
						UKBB	0.984	0.899	1.076	0.734	0.0	
						Stage 2	0.970	0.917	1.026	0.295	0.0	
rs3750904	2	167055393	T	C	0.996	Stage 1	0.859	0.641	1.154	0.323	0.0	*FXN*
						UKBB	0.791	0.513	1.217	0.291		
						Stage 2	0.859	0.640	1.154	0.325	0.0	
rs2026739	9	32418237	G	T	0.288	Stage 1	1.026	0.985	1.069	0.227	0.6	*ACO1*
						UKBB	1.001	0.949	1.056	0.960		
						Stage 2	1.017	0.984	1.050	0.325	0.0	
rs270388	6	7772340	T	G	0.159	Stage 1	0.991	0.939	1.044	0.730	0.5	*BMP6*
						UKBB	0.962	0.901	1.028	0.261		
						Stage 2	0.980	0.940	1.021	0.339	0.4	
rs2284017	22	37096927	T	C	0.436	Stage 1	1.006	0.968	1.046	0.749	0.3	*CACNG2*
						UKBB	0.957	0.911	1.004	0.072		
						Stage 2	0.987	0.958	1.017	0.399	0.4	
rs8007201	14	55324848	A	G	0.669	Stage 1	1.002	0.963	1.043	0.909	0.4	*GCH1*
						UKBB	1.032	0.981	1.087	0.221		
						Stage 2	1.014	0.982	1.046	0.404	0.3	
rs480760	3	195798258	T	C	0.036	Stage 1	1.001	0.906	1.106	0.983	0.0	*TFRC*
						UKBB	0.917	0.807	1.042	0.190		
						Stage 2	0.968	0.895	1.048	0.435	0.0	
rs13075921	3	148915628	T	C	0.896	Stage 1	1.011	0.947	1.079	0.747	0.0	*CP*
						UKBB	0.939	0.868	1.017	0.121		
						Stage 2	0.982	0.934	1.032	0.475	0.0	
rs927312	6	8559593	G	C	0.868	Stage 1	0.981	0.925	1.039	0.502	0.0	*HLA-DQB1*03:02*
						UKBB	1.070	0.997	1.149	0.059		
						Stage 2	1.015	0.971	1.062	0.515	0.2	
rs224446	12	51381718	C	T	0.849	Stage 1	1.001	0.953	1.052	0.898	0.04	*SLC11A2*
						UKBB	0.958	0.896	1.025	0.210		
						Stage 2	0.986	0.946	1.028	0.517	0.3	
rs3816893	3	148927711	A	T	0.900	Stage 1	0.990	0.928	1.057	0.771	0.0	*CP*
						UKBB	0.974	0.899	1.055	0.512		

© 2021 Veluchamy A et al. *JAMA Network Open.*

RSID	CHR	POS	EA	NEA	EAF	Study	OR	OR_95L	OR_95U	P_{unadj}	I^2	GENE
						Stage 2	0.984	0.935	1.034	0.530	0.0	
						Stage 1	0.993	0.950	1.038	0.764	0.0	
						UKBB	1.037	0.982	1.095	0.195		
rs11674595	2	102610992	T	C	0.736	Stage 2	1.010	0.976	1.045	0.572	0.0	*IL1R2*
						Stage 1	1.004	0.962	1.048	0.838	0.0	
						UKBB	1.018	0.965	1.074	0.523		
rs10098807	8	21708824	G	A	0.719	Stage 2	1.010	0.977	1.044	0.579	0.0	*GFRA2*
						Stage 1	1.141	0.936	1.392	0.200	0.0	
						UKBB	0.922	0.736	1.157	0.481		
rs13306435	7	22771039	T	A	0.989	Stage 2	1.040	0.896	1.208	0.611	0.1	*IL6*
						Stage 1	1.002	0.960	1.045	0.933	0.0	
						UKBB	1.018	0.965	1.073	0.520		
rs17428041	8	21711431	T	C	0.719	Stage 2	1.008	0.975	1.042	0.648	0.0	*GFRA2*
						Stage 1	1.014	0.976	1.055	0.479	0.0	
						UKBB	0.995	0.947	1.045	0.842		
rs267202	6	7854236	G	A	0.615	Stage 2	1.007	0.977	1.038	0.668	0.0	*BMP6*
						Stage 1	0.996	0.671	1.300	0.692	0.0	
						UKBB	1.054	0.615	1.806	0.851		
rs74449889	2	167160735	A	G	0.996	Stage 2	0.934	0.671	1.300	0.693	NA	*SCN9A*
						Stage 1	0.992	0.918	1.074	0.863	0.0	
						UKBB	1.042	0.942	1.151	0.423		
rs4866176	5	20245554	C	T	0.939	Stage 2	1.011	0.951	1.076	0.729	0.0	*CDH18*
						Stage 1	1.005	0.914	1.105	0.919	0.1	
						UKBB	1.025	0.908	1.157	0.680		
rs3793451	9	71659280	C	T	0.958	Stage 2	1.013	0.940	1.091	0.745	0.1	*FXN*
						Stage 1	0.995	0.952	1.040	0.841	0.0	
						UKBB	1.022	0.967	1.081	0.431		
rs11780601	8	21717841	G	T	0.751	Stage 2	1.006	0.972	1.041	0.749	0.0	*GFRA2*
						Stage 1	1.025	0.977	1.075	0.321	0.0	
						UKBB	0.977	0.921	1.037	0.453		
rs3783641	14	55360139	T	A	0.797	Stage 2	1.006	0.969	1.044	0.753	0.0	*GCH1*
						Stage 1	1.021	0.961	1.084	0.502	0.1	
						UKBB	0.983	0.915	1.057	0.654		
rs1799971	6	154360797	A	G	0.874	Stage 2	1.006	0.960	1.053	0.819	0.1	*OPRM1*
						Stage 1	1.002	0.954	1.051	0.943	0.2	
						UKBB	1.007	0.948	1.069	0.810		
rs1518111	1	206944645	T	C	0.199	Stage 2	1.004	0.967	1.042	0.839	0.0	*IL10*
rs1883988	22	37105180	G	A	0.740	Stage 1	0.971	0.927	1.013	0.179	0.0	*CACNG2*

© 2021 Veluchamy A et al. *JAMA Network Open.*

RSID	CHR	POS	EA	NEA	EAF	Study	OR	OR_95L	OR_95U	P_{unadj}	I^2	GENE
						UKBB	1.056	0.999	1.115	0.054		
						Stage 2	1.003	0.969	1.038	0.872	0.5	
						Stage 1	0.999	0.952	1.049	0.988	0.2	
						UKBB	1.008	0.949	1.070	0.791		
rs1518110	1	206944861	A	C	0.199	Stage 2	1.003	0.966	1.041	0.883	0.0	*IL10*
						Stage 1	1.004	0.967	1.042	0.832	0.0	
						UKBB	0.988	0.942	1.037	0.641		
rs1800896	1	206946897	T	C	0.489	Stage 2	0.998	0.969	1.028	0.905	0.0	*IL10*
						Stage 1	1.004	0.963	1.047	0.848	0.0	
						UKBB	0.998	0.947	1.052	0.951		
rs12596162	16	87151495	C	T	0.691	Stage 2	1.002	0.970	1.035	0.909	0.0	*PRKCA*
						Stage 1	0.989	0.945	1.036	0.660	0.0	
						UKBB	1.013	0.953	1.076	0.691		
rs3917332	2	102796524	A	T	0.197	Stage 2	0.998	0.962	1.035	0.912	0.0	*IL1R1*
						Stage 1	1.004	0.967	1.042	0.826	0.1	
						UKBB	0.999	0.942	1.037	0.643		
rs1878672	1	206943713	G	C	0.489	Stage 2	0.998	0.969	1.028	0.913	0.0	*IL10*
						Stage 1	1.002	0.957	1.050	0.917	0.0	
						UKBB	0.999	0.942	1.060	0.991		
rs752688	14	55311569	C	T	0.789	Stage 2	1.001	0.965	1.039	0.941	0.0	*GCH1*
						Stage 1	1.002	0.956	1.050	0.917	0.1	
						UKBB	0.999	0.942	1.059	0.970		
rs4411417	14	55320563	T	C	0.789	Stage 2	1.001	0.965	1.038	0.953	0.0	*GCH1*
						Stage 1	0.942	0.835	1.063	0.346	0.0	
						UKBB	1.096	0.939	1.278	0.251		
rs2718796	3	133479200	G	C	0.025	Stage 2	0.998	0.908	1.097	0.966	0.0	*TF*
						Stage 1	1.005	0.967	1.043	0.803	0.0	
						UKBB	0.991	0.944	1.039	0.712		
rs3024496	1	206941864	A	G	0.490	Stage 2	0.999	0.970	1.030	0.972	0.0	*IL10*
						Stage 1	0.999	0.953	1.046	0.956	0.1	
						UKBB	1.001	0.944	1.062	0.971		
rs10483639	14	55306457	G	C	0.788	Stage 2	1.000	0.964	1.037	0.986	0.0	*GCH1*
						Stage 1	0.972	0.929	1.015	0.211	0.5	
						UKBB	1.045	0.989	1.103	0.123		
rs2284015	22	37096573	C	G	0.741	Stage 2	1.000	0.966	1.035	0.991	0.6	*CACNG2*
						Stage 1	1.012	0.972	1.053	0.558	0.1	
						UKBB	0.981	0.934	1.031	0.462		
rs4820242	22	36982675	G	A	0.385	Stage 2	1.000	0.970	1.031	0.1	0.1	*CACNG2*

© 2021 Veluchamy A et al. *JAMA Network Open.*

eTable 8. Combined Analysis of the Present Study Summary Statistics (Stage 1 and Stage 2) and the Original Study Summary Statistics

**SNV**	**Candidate gene**	**Effect allele (EAF)**	**Stage 1 (GoDARTS + GS:SFHS)** **OR (95% CI) / P_{unadj}**	**Previously reported statistics (Kallianpur et al. 2014)** **OR (95% CI) / P_{unadj}**	**Stage 1 meta-analysis + previous study)** **OR (95% CI) / P_{unadj}**	**UKBB** **OR (95% CI) / P_{unadj}**	**Stage 2 meta-analysis** **OR (95%CI) / P_{unadj}**	**Stage 2 meta-analysis + previous study** **OR (95%CI) / P_{unadj}**
**rs1901531**	*B2M*	C (0.19)	1.02(0.98-1.07) /**0.023**	1.60(1.06-2.41) /**0.028**	1.07(1.02-1.13) /**0.004**	1.00(0.94-1.07)/0.95	1.01(1.00-1.08) /**0.040**	**1.04(1.00-1.09) /0.028**
**rs7033149**	*ACO1*	G (0.15)	1.069(1.014-1.128) /**0.015**	1.60(1.11-2.40) /**0.012**	1.08(1.03-1.14) /**0.004**	0.98(0.92-1.05)/0.62	1.03(0.99-1.08)/0.111	1.04(1.00-1.08) /0.071

EAF, effect allele frequency; OR, odds ratio; OR_95L, 95% lower confidence interval; OR_95U, 95% upper confidence interval; GoDARTS, Genetics of Diabetes Audit and Research in Tayside Scotland; GS:SFHS, Generation Scotland: Scottish Family Health Study; UKBB, United Kingdom Biobank.

© 2021 Veluchamy A et al. *JAMA Network Open.*

eFigure 1. Quantile-Quantile Plot for the Results of Stage 2 Meta-analysis (GoDARTS, GS:SFHS, and UKBB) GWAS

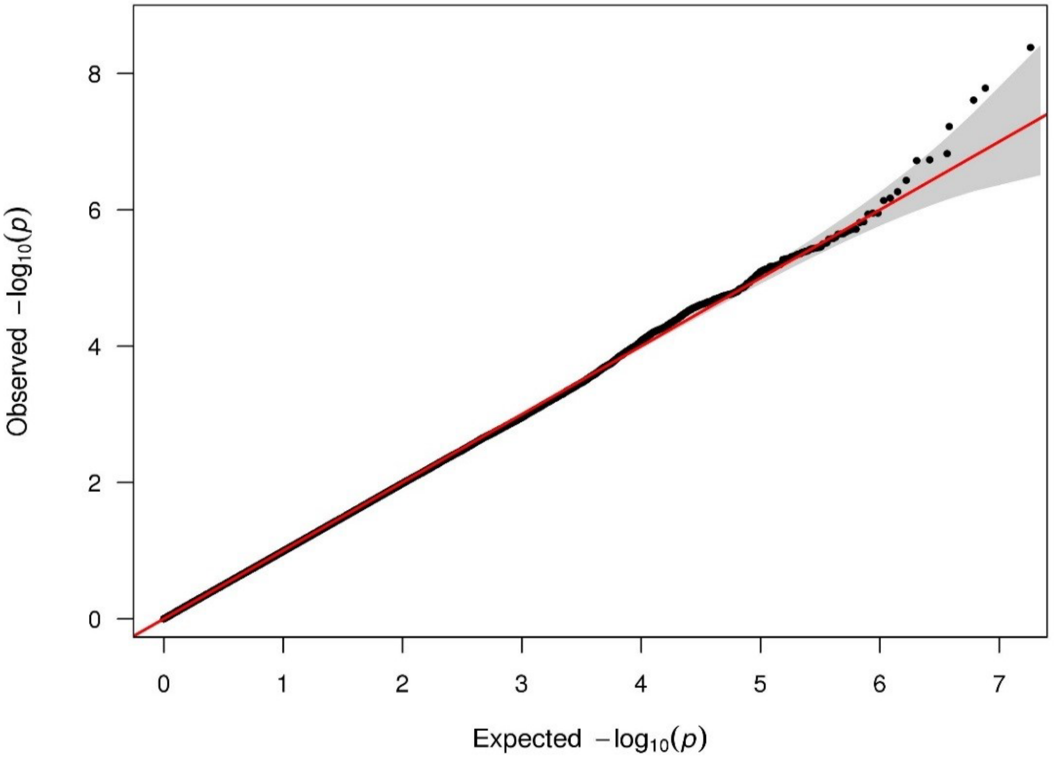

X-axis represents –log10 expected P-values and Y-axis represents –log10 observed P-values.

© 2021 Veluchamy A et al. *JAMA Network Open.*

eFigure 2. Scattershot of Directly Typed Variant at Chromosome 12q23.1 From the UKBB Data

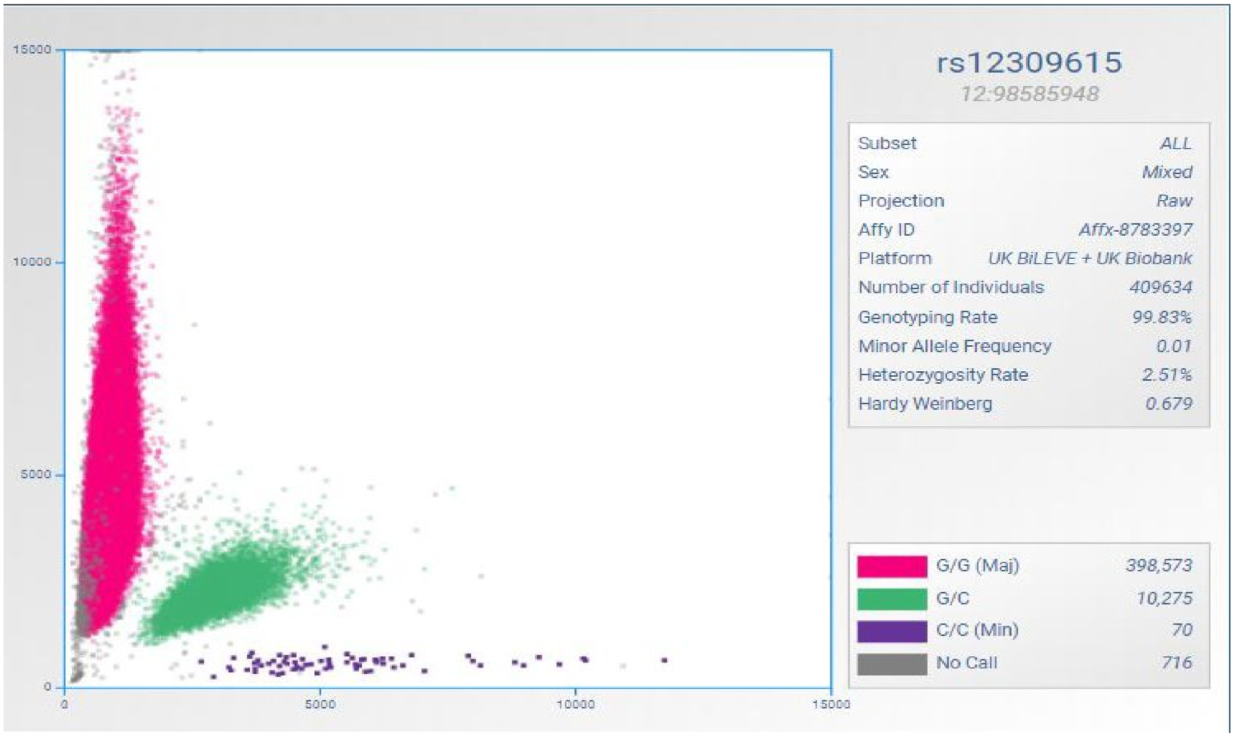

© 2021 Veluchamy A et al. *JAMA Network Open.*

eFigure 3. Multitissue eQTL Comparison for a Promising Candidate SNV for NP and Correlation With Expression of *CAB39L*

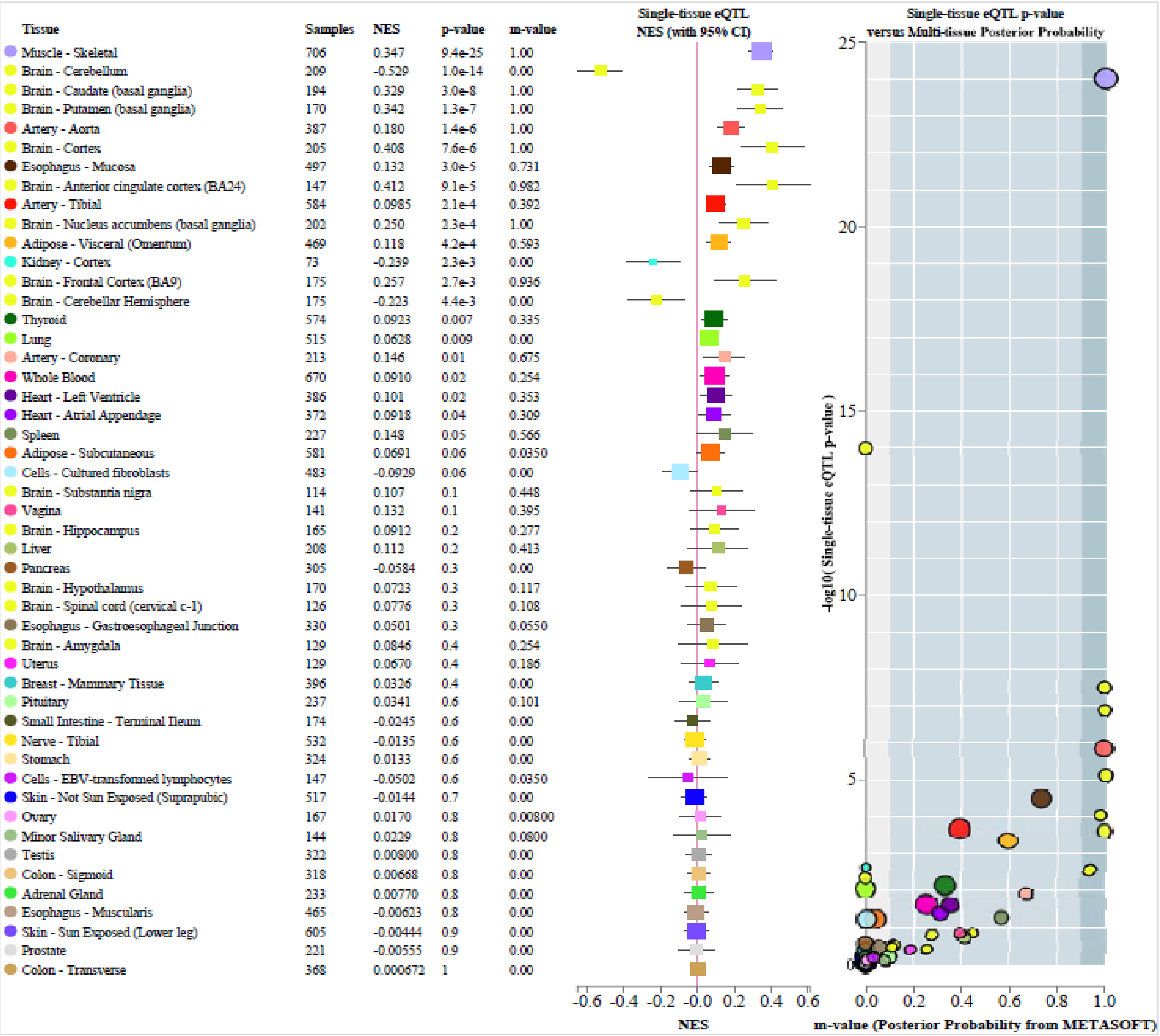

© 2021 Veluchamy A et al. *JAMA Network Open.*

eFigure 4. Plots Showing the P Value of Association Tests for SNVs With Possible NP in Stage 1 Meta-analysis (GoDARTS and GS:SFHS)

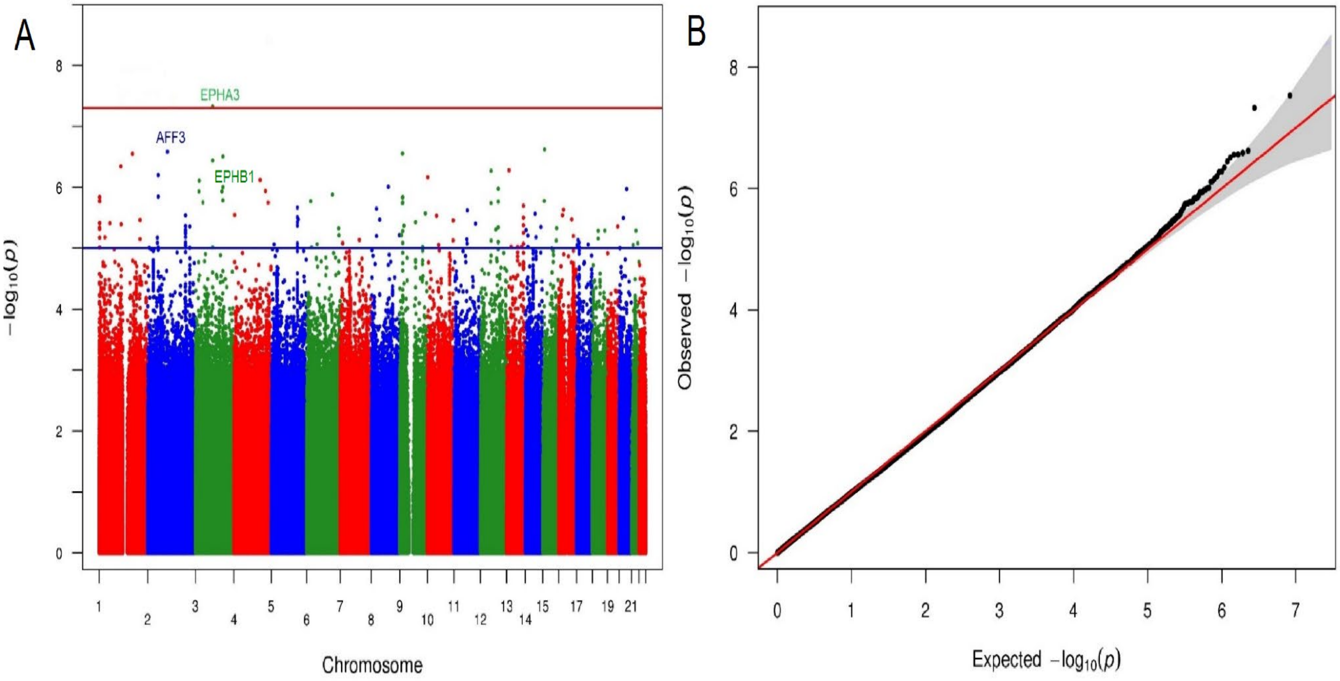

A) Manhattan plot and B) Quantile-Quantile plot.

© 2021 Veluchamy A et al. *JAMA Network Open.*

eFigure 5. *Regional Association Plot of an Index SNV in Stage 1 Meta-analysis (GoDARTS and GS:SFHS)*

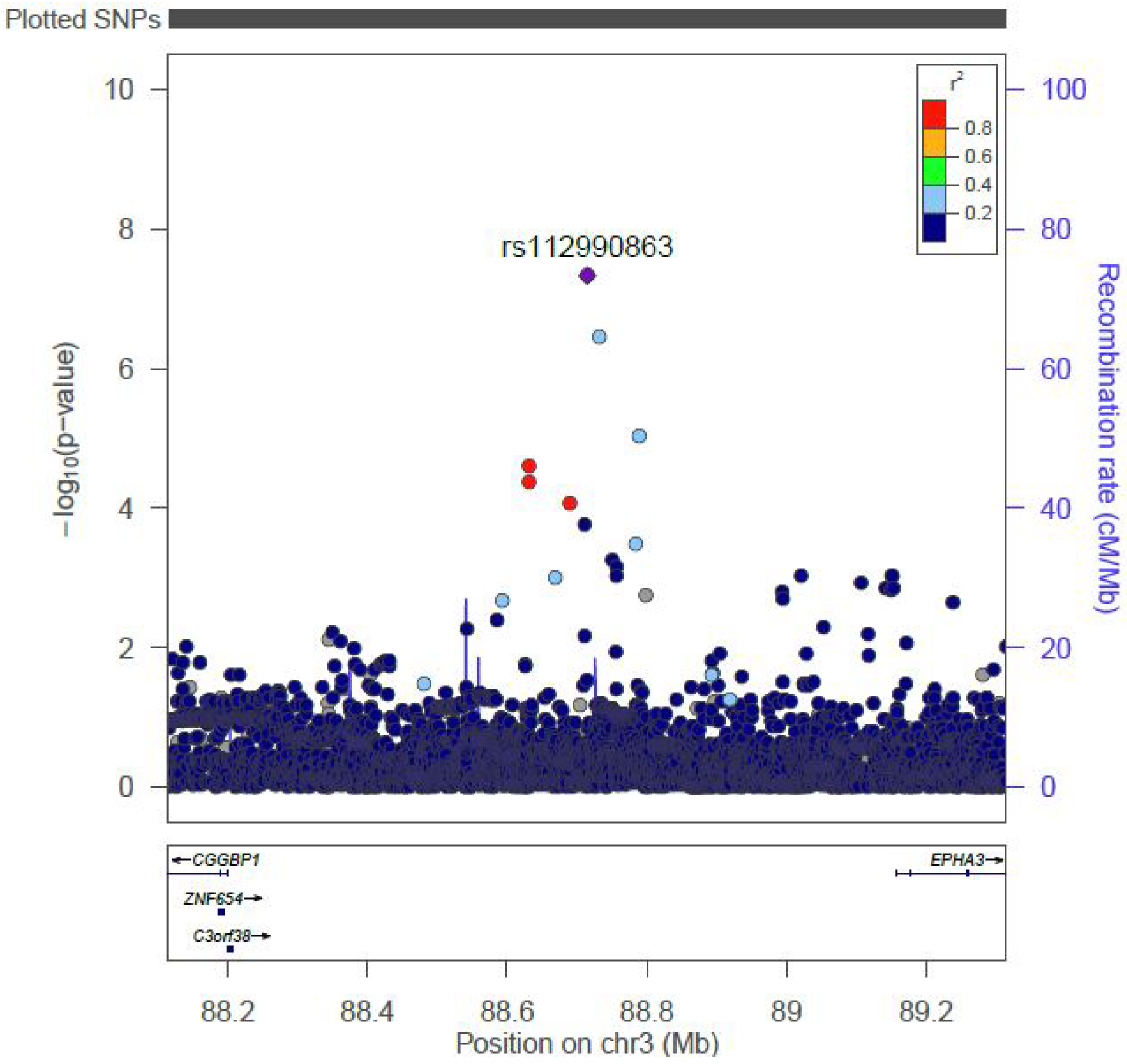

© 2021 Veluchamy A et al. *JAMA Network Open.*
